# A Fully Automated Self-help Biopsychosocial Transdiagnostic Digital Intervention to Reduce Anxiety and/or Depression and Improve Emotional Regulation and Well-being: Pre–Follow-up Single-Arm Feasibility Trial

**DOI:** 10.2196/43385

**Published:** 2023-05-30

**Authors:** Britt Klein, Huy Nguyen, Suzanne McLaren, Brooke Andrews, Kerrie Shandley

**Affiliations:** 1 Health Innovation & Transformation Centre Federation University Australia Ballarat Australia; 2 Biopsychosocial and eHealth Research & Innovation Hub Federation University Australia Ballarat Australia; 3 Charles Sturt University Port Macquarie Australia

**Keywords:** anxiety, depression, fully automated, self-help, digital intervention, transdiagnostic, biopsychosocial, emotion regulation, allostatic load, brain plasticity, positive affect, comorbidity

## Abstract

**Background:**

Anxiety disorders and depression are prevalent disorders with high comorbidity, leading to greater chronicity and severity of symptoms. Given the accessibility to treatment issues, more evaluation is needed to assess the potential benefits of fully automated self-help transdiagnostic digital interventions. Innovating beyond the current transdiagnostic one-size-fits-all shared mechanistic approach may also lead to further improvements.

**Objective:**

The primary objective of this study was to explore the preliminary effectiveness and acceptability of a new fully automated self-help biopsychosocial transdiagnostic digital intervention (Life Flex) aimed at treating anxiety and/or depression, as well as improving emotional regulation; emotional, social, and psychological well-being; optimism; and health-related quality of life.

**Methods:**

This was a real-world pre-during-post-follow-up feasibility trial design evaluation of Life Flex. Participants were assessed at the preintervention time point (week 0), during intervention (weeks 3 and 5), at the postintervention time point (week 8), and at 1- and 3-month follow-ups (weeks 12 and 20, respectively).

**Results:**

The results provided early support for the Life Flex program in reducing anxiety (Generalized Anxiety Disorder 7), depression (Patient Health Questionnaire 9), psychological distress (Kessler 6), and emotional dysregulation (Difficulties in Emotional Regulation 36) and increasing emotional, social, and psychological well-being (Mental Health Continuum—Short Form); optimism (Revised Life Orientation Test); and health-related quality of life (EQ-5D-3L Utility Index and Health Rating; all false discovery rate [FDR]<.001). Large within-group treatment effect sizes (range |*d*|=0.82 to 1.33) were found for most variables from pre- to postintervention assessments and at the 1- and 3-month follow-up. The exceptions were medium treatment effect sizes for EQ-5D-3L Utility Index (range Cohen *d*=−0.50 to −0.63) and optimism (range Cohen *d*=−0.72 to −0.79) and small-to-medium treatment effect size change for EQ-5D-3L Health Rating (range Cohen *d*=−0.34 to −0.58). Changes across all outcome variables were generally strongest for participants with preintervention clinical comorbid anxiety and depression presentations (range |*d*|=0.58 to 2.01) and weakest for participants presenting with nonclinical anxiety and/or depressive symptoms (|*d*|=0.05 to 0.84). Life Flex was rated as acceptable at the postintervention time point, and participants indicated that they enjoyed the transdiagnostic program and biological, wellness, and lifestyle-focused content and strategies.

**Conclusions:**

Given the paucity of evidence on fully automated self-help transdiagnostic digital interventions for anxiety and/or depressive symptomatology and general treatment accessibility issues, this study provides preliminary support for biopsychosocial transdiagnostic interventions, such as Life Flex, as a promising future mental health service delivery gap filler. Following large-scale, randomized controlled trials, the potential benefits of fully automated self-help digital health programs, such as Life Flex, could be considerable.

**Trial Registration:**

Australian and New Zealand Clinical Trials Registry ACTRN12615000480583; https://www.anzctr.org.au/Trial/Registration/TrialReview.aspx?id=368007

## Introduction

### Background

Anxiety disorders (ADs) and depression are highly prevalent emotional disorders [1] that cause considerable disability and societal costs [2,3], with <50% of people experiencing these disorders accessing support [4,5]. Comorbidity rates are high within ADs and between ADs and depression (ranging between 40% and 80%) [6]. Furthermore, comorbidities are associated with greater chronicity, severity, and a more complicated clinical course [6-8].

Frontline treatments for ADs and/or depression include face-to-face psychological therapy, pharmacotherapy, or a combination of these. A large body of evidence demonstrates that anxiety and depression can be effectively treated using cognitive behavioral therapy (CBT). Meta-analytic studies indicate that face-to-face CBT yields uncontrolled effect sizes of around 0.90 for various ADs [9] and 0.82 for depression [10].

Research also suggests that ADs and depression have the same underlying transdiagnostic structure, described by the *internalizing-externalizing model* [11], that informs treatment. A large amount of growing evidence indicates that transdiagnostic treatments (treatments that simultaneously treat more than one disorder, such as ADs and depression) are effective. For example, a recent meta-analytic study found similar effects for disorder-specific interventions (Cohen *d*=0.95) and transdiagnostic interventions (Hedges *g*=1.06) on anxiety measures [12].

To date, transdiagnostic programs for ADs and/or depression have largely used either a more traditional CBT framework or the Unified Treatment Protocol (UP) for the emotional disorders [13-15]. Transdiagnostic programs are also characterized as being either theory based (one size fits all) or tailored (individualizing a treatment to a specific person based on their unique comorbidities). The defining feature of theory-based transdiagnostic treatments (sometimes called shared mechanistic transdiagnostic interventions) is that they are developed to target the common processes (or mechanisms) that underlie the development and maintenance of emotional disorders [16]. The main foci of transdiagnostic CBT interventions are on changing psychopathological cognition and behaviors, and the UP additionally considers the role of negative emotions (teaching people to become more aware of, and better regulate, their negative emotions) when treating anxiety and depression.

Although disorder-specific CBT and transdiagnostic interventions have traditionally been delivered face-to-face, they have also been administered on the web. Given treatment barriers, such as cost, stigma, long waitlists, travel or convenience, and preference for self–management-oriented delivery models of care, digitally based mental health interventions have provided greater treatment choices [17] and are a cost-effective alternative [18] to disorder-specific and transdiagnostic treatments delivered face-to-face. Digital translations of largely single-disorder–focused CBT-based psychological treatments have been established as an effective alternative to face-to-face delivery [19,20]. For example, a meta-analytic study by Andrews et al [20] on web-based interventions for depression or one of the ADs yielded a controlled effect size of 0.88. Furthermore, a recent meta-analytic study found that therapist-assisted transdiagnostic digital treatments for anxiety and depression yielded uncontrolled effect sizes of 0.96 for both depression and anxiety outcomes [21].

However, the bulk of transdiagnostic digital interventions evaluated to date have involved some kind of therapist support and/or researcher contact (eg, to conduct clinical assessments). Although the inclusion of a therapist (or researcher contact) has been found to increase adherence to digital intervention programs, it is also more costly to operate and far less scalable [19]. Given the high prevalence and associated disability of ADs and/or depression and the limited resources available to treat every person with such disorders, evaluating the effectiveness and acceptability of fully automated self-help transdiagnostic digital interventions is timely.

At present, there appears to be four key areas to explore further when it comes to digital transdiagnostic programs: (1) testing fully automated self-help transdiagnostic versions, (2) testing transdiagnostic comorbidity effects, (3) testing transdiagnostic preventative effects, and (4) exploring the expansion of current transdiagnostic intervention content based on scientific advancements.

First, to date, almost no studies have investigated the potential effectiveness of a fully automated self-help transdiagnostic digital intervention for anxiety and/or depression symptoms (ie, those with no direct contact with a therapist and/or researcher). The only published study found was by Batterham et al [22], which evaluated their FitMindKit video-based CBT transdiagnostic web-based program. Significant effects were found on depressive, panic, and/or anxiety symptoms relative to an attention control condition from pre- to postintervention periods (over 4 weeks). However, although the Batterham et al [22] trial was a large (N=1986) and robust study including the attention control condition, there was no follow-up assessment following the postintervention assessment.

Second, although there have been dozens of studies evaluating transdiagnostic digital interventions, their impact on improving comorbidity symptoms has received far less examination [23,24]. For example, FitMindKit evaluation by Batterham et al [22] was one of many transdiagnostic studies that did not directly assess any comorbidity intervention outcome effects. Given that the core function of a transdiagnostic program is to simultaneously treat different disorders, investigating comorbidity intervention effects, where possible, is much needed.

Third, in relation to the potential preventative effects of transdiagnostic interventions, people with nonclinical subthreshold anxiety and/or depressive symptom levels are typically left untreated despite the experience of some impairment, distress, and potential worsening of symptoms [17]. Given the risk that these people can have in developing a clinical form of emotional disorders, offering people early intervention via transdiagnostic treatment protocols (especially via self-help formats) could be of benefit by acting as an indicated prevention activity and therefore should be explored further [22,25]. To date, 1 group [25] has reported on the preventative effects of a transdiagnostic digital program (with therapist assistance) on adolescents (N=30). Schmitt et al [25] found that symptoms of self-reported anxiety and depression, clinician-rated symptom severity, and self-reported and parent-reported severity of the main problems had significantly improved over time; however, further investigation was required.

Fourth, in reference to expanding transdiagnostic program offerings, although UP considers cognitive, behavioral, and emotional mechanisms to underlie emotional disorders, it does not include teachings around increasing positive affect (rather it focuses on reducing negative affect or deficits). To date, 1 group [26] has extended their CBT+Emotional Regulation transdiagnostic digital program by adding content and strategies to specifically enhance positive affect. When comparing their transdiagnostic intervention plus positive affect–based modules to a version without the positive affect modules, they found consistently larger treatment effect sizes for the transdiagnostic intervention plus a positive affect module extension program relative to their standard transdiagnostic protocol (although no significant difference was found between the 2 conditions).

Furthermore, given the recent theory formulation and evidence around the links between chronic stress and the development and maintenance of anxiety and/or depression [27,28], it may prove beneficial to include coverage around allostasis (the biological mechanism behind the deactivation of the stress response) [29,30]; heart rate variability (a psychophysiological marker of mental and physical well-being) [31,32]; and allostatic load (being the regular activation of the stress response and/or not managing the deactivation of the stress response, which can lead to poor psychological and physical ill health effects because of stress response activation wear and tear on the body) [27,30,33].

Nonpharmacological biologically focused interventions (ie, lifestyle-based interventions that work on enabling brain plasticity; increase heart rate variability; and decrease allostatic load, such as increasing physical activity, brain and gut nutrition, mindfulness, and sleep hygiene) are showing promise in reducing anxiety and depressive symptoms [31,34-37] and could serve as an adjuvant therapy to more traditional CBT and UP strategies (eg, breathing control, progressive muscle relaxation, cognitive appraisal, acceptance techniques, and emotional regulation strategies). Consideration could also be given to explaining the concept of brain or neural plasticity [38-40] within the transdiagnostic intervention. This concept can offer a more concrete explanation of the possibility of change (biologically, cognitively, emotionally, and behaviorally) and how such changes occur.

### Objective

The primary aim of this study was to explore the feasibility of a new fully automated self-help biopsychosocial transdiagnostic digital intervention (Life Flex), which aims to treat anxiety and/or depression, as well as improve emotional regulation; emotional, social, and psychological well-being; optimism; and health-related quality of life by measuring possible intervention outcome changes over time. The secondary aim of this study was to consider any potential comorbidity and preventative intervention effects of the Life Flex program, and the third aim was to explore participants’ quantitative and qualitative responses around the acceptability of the Life Flex intervention, including the expansion of the biologically focused and positive affect content.

Our primary exploratory questions were as follows: (1) Do participants show significant decreases in measures of anxiety and depression (primary outcomes) from preintervention to postintervention and at the 1- and 3-month follow-up assessments? (2) Do participants show improvements in emotional regulation; psychological distress; emotional, social, and psychological well-being; optimism; and health-related quality of life from preintervention to postintervention and at the 1- and 3-month follow-up assessments? And (3) Do participants rate the Life Flex program as acceptable?

## Methods

### Participants

Participants were recruited via health websites. Inclusion criteria were that the participants must be aged ≥18 years, have interest in undertaking a mental health digital intervention focused on anxiety and/or depressive symptoms, have internet access, and provide web-based informed consent. Of the 347 initial registrations, 79 (22.8%) participants did not complete the preassessment questionnaires (n=64, 81% signed up and did not return to complete the preassessment questionnaires; and n=15, 19% of participants started the preassessment questionnaires but did not complete it), leaving 268 (77.2%) study participants. However, 27 (10.1%) of the 268 participants were later removed, as they self-identified as being nongenuine program participants (eg, health care practitioners interested in the program for their patients and IT developers wanting to see the program). The final sample consisted of 241 adults. Following the completion of the digital preintervention assessment questionnaires, participants were immediately given access to the Life Flex digital health program.

### Design

Life Flex was tested using a pre-during-post-follow-up single-arm feasibility trial design. Data were collected at week 0 (preintervention period), weeks 3 and 5 of the intervention (during intervention assessments), the postintervention period (week 8), and 1-month (week 12) and 3-month (week 20) follow-ups. This study reported the outcome data collected at all time points.

### Ethics Approval

This study was approved by the Federation University Human Research Ethics Committee (approval A15-005) and was preregistered with the Australian and New Zealand Clinical Trials Registry (ACTRN12615000480583). All participants gave their consent to participate in the study by reading a Plain Language Information Statement on the web and clicking a checkbox stating that “I have read the Plain Language Information Statement and I agree to the above conditions.” All data were deidentified before analysis.

### Intervention

Life Flex was specifically designed to provide people with information and strategies to address their anxiety and/or depressive symptoms and contains 6 *core* modules, plus an Introduction module delivered over 7 weeks. Each module takes approximately 25 minutes to complete. In addition, to reinforce the module-based information, there are 20 to 30 minutes of offline activities each week. Offline activities include applying the concepts and techniques discussed in the modules such as self-monitoring depressive and anxiety symptoms, undertaking one of the increasing biological and wellness flexibility intervention strategies, monitoring emotions and thoughts, and undertaking a gradual exposure or behavioral activation activity. Participants also received various automated emails (eg, to remind them to log on and when to complete the intervention assessments). Modules include text, graphics, audio, video, editable forms, interactive quizzes and games (eg, brain training), and downloads. The intervention is accessible via web, mobile, or tablet devices, and module release used a stage-release design (sequential release of each module following the completion of the previous one). Participants could continue to use the Life Flex program outside of scheduled assessments over a 20-week period. A summary of these modules is provided in Multimedia Appendix 1.

### Measures

#### Outcome Measures

Web-based questionnaires were scheduled at the preintervention time point (week 0), postintervention time point (week 8), and 1-month (week 12) and 3-month (week 20) follow-ups, except for the Generalized Anxiety Disorder 7 (GAD-7) and Patient Health Questionnaire 9 (PHQ-9), which were also administered during the intervention (weeks 3 and 5).

#### Exploration of Preliminary Effectiveness

##### Primary Outcome Measures

The *GAD-7* [41] is a 7-item scale, with scores ranging from 0 to 21, and is used to measure generalized anxiety, with higher scores reflecting higher anxiety, using a clinical cutoff of 8. The *PHQ-9* [42] is a 9-item scale, with scores ranging from 0 to 27, and is used to measure depression, with higher scores reflecting higher depressive symptoms, with a clinical cutoff of 10.

##### Secondary Outcome Measures

The *Kessler 6* (K-6) [43] is a 6-item scale, with scores ranging from 10 to 30, and is used to measure psychological distress, with higher scores reflecting higher distress, with scores between 10 and 15 categorized as “Likely to be well.” The *Difficulties in Emotional Regulation 36* (DERS-36) [44] is a 36-item scale, with scores ranging from 36 to 180, and is used to measure emotional regulation, with higher scores reflecting greater levels of dysregulation. The *Mental Health Continuum—Short Form* (MHC-SF) [45] is a 14-item scale, with scores ranging from 0 to 70, and is used to measure emotional, social, and psychological well-being, with higher scores reflecting greater well-being. The *Revised Life Orientation Test* (R-LOT) [46] is a 10-item scale, with scores ranging from 0 to 24, and is used to measure optimism, with higher scores reflecting higher optimism. The EQ-5D-3L [47] is a 5-item scale, with scores converted to a utility index score (EQ-5D-3L Utility Index), and is used to measure quality of life, with lower utility index scores reflecting poorer quality of life. The sixth question asks the person to rate their current health state (EQ-5D-3L Health Rating) from 0 to 100, with higher ratings reflecting greater health.

#### Acceptability

The *Treatment Acceptability and Satisfaction Questionnaire* (TAS-Q; self-developed) was used to measure participants’ satisfaction with the intervention at the postintervention time point. Eight single-item multiple-choice questions were asked regarding program acceptability and credibility (eg, How would you rate the quality of the Life Flex program?) and satisfaction (eg, In an overall, general sense, how satisfied are you with the Life Flex program?). Each item used a 4-point (eg, *Yes, definitely*; *Yes, generally*; *No, not really*; and *No, definitely not*) or 5-point (*Very satisfied*, *Satisfied*, *Neutral*, *Dissatisfied*, and *Very dissatisfied*; or *Very Poor*, *Poor*, *Fair*, *Good*, and *Excellent*) Likert scale. There were also open-text response questions asking participants what they believed were the best and worst parts of the Life Flex digital health program.

In addition, a 7-item *Treatment Expectancy and Credibility/Acceptability Scale* [48] was administered at the preintervention time point, with a total score ranging from 0 to 70 and higher scores indicating higher intervention expectancy and credibility and acceptability. A range of single-item questions were asked to gather participant characteristics, for example, age, gender, income, sexual orientation, alcohol and drug use, current panic disorder, social AD, specific phobia, posttraumatic stress disorder, and obsessive-compulsive disorder symptoms.

### Statistical Analysis

#### Overview

Multimedia Appendix 2 shows the missing data patterns across the intervention program timeline. Given the considerable missing data pattern, we opted not to use multiple imputations. However, our available data suggest that they would represent the entire sample of the study (*Sensitivity Analysis* section).

#### Exploration of Preliminary Intervention Effects

All analyses were performed using Stata (version 17; StataCorp) and SPSS (version 29; IBM Corp). Categorical data were presented as numeric and percentage forms, and continuous data were presented as mean (SD) where relevant. Independent 2-tailed *t* tests and chi-square tests (Multimedia Appendix 3) were used to compare the mean difference between the groups (complete data set vs missing data set grouping). ANOVAs (Multimedia Appendix 4) were used to compare the mean difference between preintervention clinical diagnostic presentation subgroups (anxiety, depression, comorbid, and nonclinical) on the baseline outcome measures. Categorical variables related to sociodemographic characteristics (Multimedia Appendix 5) across preintervention clinical diagnostic presentation subgroups were compared by using ANOVAs and chi-square tests.

Treatment effects from preintervention to during, postintervention, and follow-up assessments were evaluated using Mann-Kendall trend statistics. Cohen *d* [49] classification scheme (small effect=0.2, medium effect=0.5, and large effect=0.8) was applied to index and interpret the standardized difference size. We adjusted for multiple testing using the false discovery rate (FDR). Here, findings with FDR<.05 were considered statistically significant. We used FDR, as our study design was explorative in nature and significant results may provide useful recommendations for future research [50-52]. Bonferroni-based adjustments are problematic in terms of irrelevant null hypothesis and increased type II error [53].

#### Diagnostic Presentation Subgroupings

Participants were categorized using the clinical disorder cutoff scores from the GAD-7 and PHQ-9. Participants scoring ≥8 on the GAD-7 and ≥10 on the PHQ-9 were categorized as having *clinical comorbid anxiety and depression*; those with a GAD-7 score of ≥8 but a PHQ-9 score <10 were categorized as having *clinical anxiety only*; those with a PHQ-9 score of ≥10 but a GAD-7 score <8 were categorized as having *clinical depression only*; and those with scores <8 on the GAD-7 and <10 on the PHQ-9 were categorized as *nonclinical anxiety and/or depression*. On classification, we continued to assess treatment effects from preintervention to during, postintervention, and follow-up assessments by subgroups using Cohen *d* metrics.

#### Sensitivity Analysis

Given that imputation for missing data was not performed, the sensitivity analysis was conducted indirectly by testing whether completer and missing data differed in terms of clinical characteristics and demographics. As there was no significant difference in demographics between the 2 subsets of data (Multimedia Appendix 3), the completer data therefore appear to adequately represent the planned sample. However, in future research, when large numbers of later time points are reached, we suggest undertaking multiple imputation using chained equations and then compare completer data and data after multiple imputation using chained equations to validate the findings.

#### Power Analysis

The required target sample size was determined using G*Power [54]. Conservatively, assuming a small-medium effect (ie, G*Power *F* test=0.25), the significance set at 5% (*P*=.05), and the power at 80%, a minimum sample of 83 was required to demonstrate statistical significance on the primary outcome measures. This number included a 50% attrition rate. However, although our initial scheduled assessment number (n=241) for preintervention assessment was much higher than required, participant completion of all the subsequent 5 scheduled assessments following preintervention assessment did not reach 83. Future research when investigating the effectiveness of a fully automated self-help version of Life Flex should bear this in mind when calculating power.

#### Attrition

**Figure 1:**
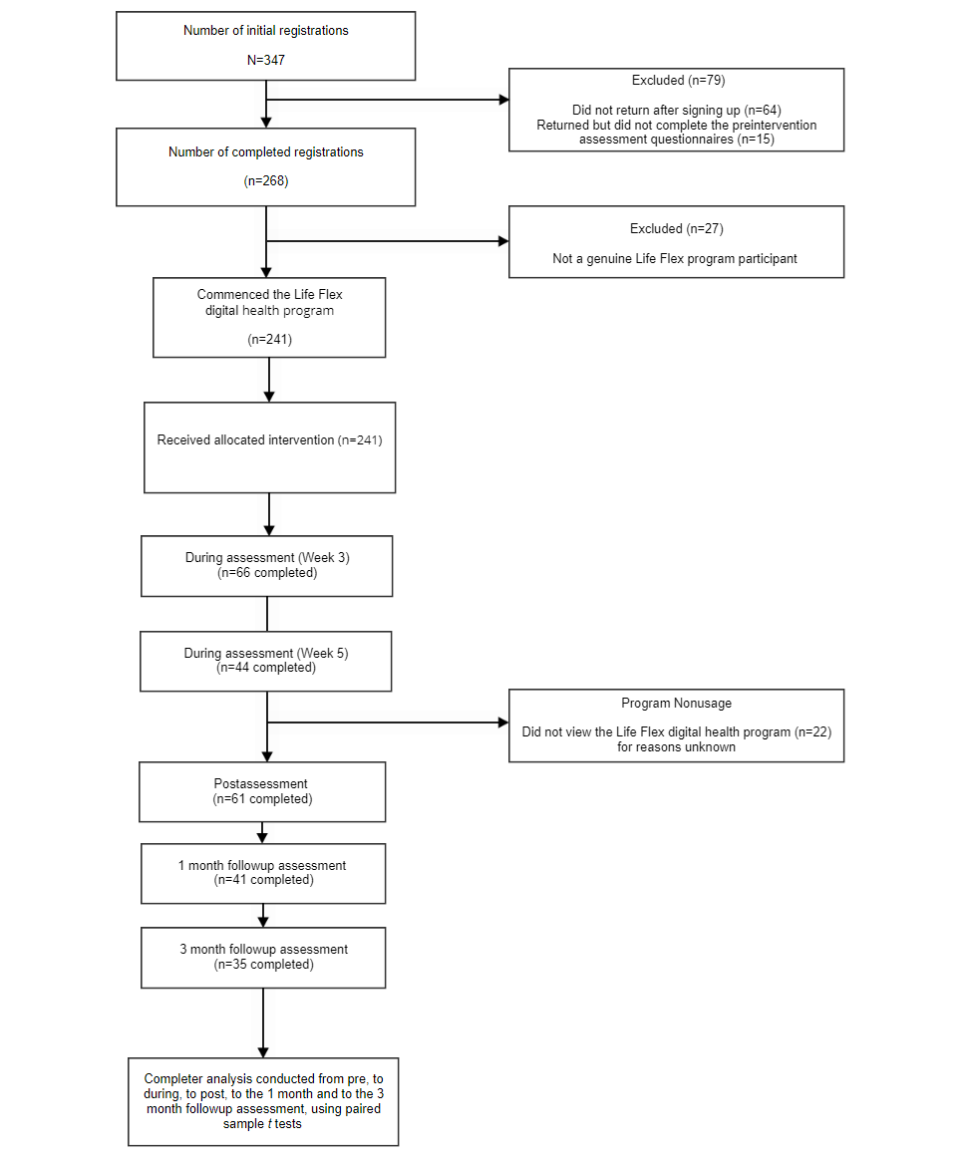
Participant study flow through trial.

In terms of attrition, 9.1% (22/241) of the participants failed to access the program modules. In terms of scheduled assessment completions, 58.1% (140/241) of the participants failed to complete at least 1 scheduled assessment following the preintervention assessment. Figure 1 shows the participant flow through the trial.

## Results

### Participant Characteristics

The average age was 43.1 (SD 13.2; range 18-79) years. Of the 241 participants involved in this study, most were female (n=156, 64.7%), were born (n=188, 78%) and residing (n=231, 95.9%) in Australia, were heterosexual (n=201, 83.4%), had obtained a bachelor’s degree (n=105, 43.6%) or higher, had a GAD-7 score≥8 (n=162, 67.2%) and PHQ-9 score≥10 (n=158, 65.6%), and had never used illicit drugs (n=163, 67.6%). Almost half of the participants (117/241, 48.5%) took psychotropic medication, whereas most (153/241, 63.5%) accessed physical health services and less than half (98/241, 40.7%) accessed mental health services in the last 4 weeks. Multimedia Appendix 6 provides the trial study participant characteristics. Participant characteristics and intervention outcomes at baseline by clinical diagnostic presentation subgroups are presented in Multimedia Appendices 4 and 5, respectively.

### Preliminary Intervention Outcome Effects

**Figure 2:**
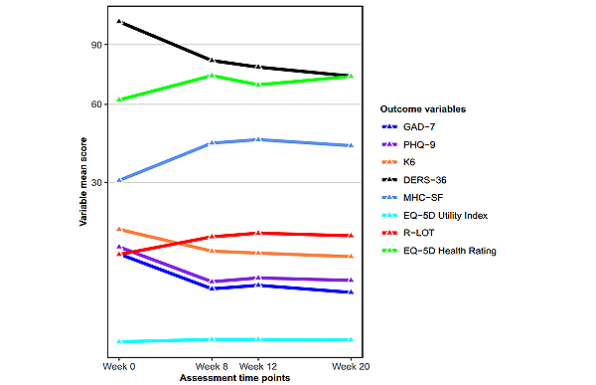
Intervention outcomes over time. DERS-36: Difficulties in Emotional Regulation 36; GAD-7: Generalized Anxiety Disorder 7; K-6: Kessler 6; MHC-SF: Mental Health Continuum—Short Form; PHQ-9: Patient Health Questionnaire 9; R-LOT: Revised Life Orientation Test.

Tables 1 and 2 display the results of the outcome measures across the scheduled assessment time points, and Figure 2 shows a visual representation of these results. Here, all 8 outcome variables showed an overall significant mean change at every assessment time point relative to their preintervention mean (FDRs remained constant compared with *P* values). The 2 primary outcome measures (GAD-7 and PHQ-9) changed from preintervention time point relative to each subsequent scheduled assessment, demonstrating a large treatment effect size change from week 5 for GAD-7 and week 8 for PHQ-9 onward (ranging from Cohen *d*=0.84 to 1.33). The secondary measures of psychological distress (K-6); emotional regulation (DERS-36); and emotional, social, and psychological well-being (MHC-SF) also showed mean changes over time, with postintervention assessment and both follow-ups (1 and 3 months) demonstrating a large treatment effect size change (ranging from |*d*|=0.82 to 1.18). The remaining secondary measures, R-LOT and quality of life (EQ-5D-3L Utility Index and Health Rating), displayed small-to-medium treatment effect size changes, ranging from Cohen *d*=−0.34 to −0.79, as well as one large treatment effect size change on the R-LOT at 1 month follow-up (Cohen *d*=−0.90).

**Table 1:** Descriptive of intervention outcomes at all scheduled assessments over time.

	Preintervention time point: week 0 (n=241), mean (SD)	During the intervention			Postintervention time point: week 8 (n=58-61), mean (SD)		1-month follow-up: week 12 (n=41), mean (SD)		3-month follow-up: week 20 (n=34-35), mean (SD)
		Week 3 (n=66), mean (SD)	Week 5 (n=44), mean (SD)						
GAD-7^a^	11.51 (5.68)	8.41 (4.84)	6.43 (3.98)	5.66 (5.01)		6.15 (5.06)		5.17 (4.87)	
PHQ-9^b^	12.94 (6.81)	9.61 (6.42)	8.55 (5.50)	6.68 (6.58)		7.27 (6.47)		6.88 (6.17)	
Kessler 6	16.88 (5.92)	N/A^c^	N/A	12.08 (5.48)		11.68 (5.04)		11.00 (5.02)	
DERS-36^d^	103.08 (25.17)	N/A	N/A	81.37 (29.44)		77.98 (28.53)		73.41 (25.54)	
MHC-SF^e^	30.61 (14.10)	N/A	N/A	43.80 (17.24)		45.17 (16.91)		42.82 (17.16)	
R-LOT^f^	11.40 (5.04)	N/A	N/A	15.15 (5.87)		16.00 (5.47)		15.38 (5.03)	
EQ-5D-3L Utility Index	0.70 (0.20)	N/A	N/A	0.82 (0.20)		0.82 (0.16)		0.80 (0.20)	
EQ-5D-3L Health Rating	62.02 (20.31)	N/A	N/A	73.67 (19.94)		69.15 (24.80)		73.26 (19.47)	

^a^GAD-7: Generalized Anxiety Disorder 7.

^b^PHQ-9: Patient Health Questionnaire 9.

^c^N/A: not applicable.

^d^DERS-36: Difficulties in Emotional Regulation 36.

^e^MHC-SF: Mental Health Continuum—Short Form.

^f^R-LOT: Revised Life Orientation Test.

**Table 2:** Change in intervention outcomes at all scheduled assessments over time.

	Week 0 vs 3, Cohen *d* (95% CI)	Week 0 vs 5, Cohen *d* (95% CI)	Week 0 vs 8, Cohen *d* (95% CI)	Week 0 vs 12, Cohen *d* (95% CI)	Week 0 vs 20, Cohen *d* (95% CI)	*P* value^a^	FDR^b^
GAD-7^c^	0.56 (0.29 to 0.84)	0.93 (0.60 to 1.26)	1.05 (0.76 to 1.35)	0.96 (0.62 to 1.30)	1.33 (0.77 to 1.50)	<.001	<.001
PHQ-9^d^	0.50 (0.22 to 0.77)	0.66 (0.34 to 0.99)	0.93 (0.63 to 1.22)	0.84 (0.50 to 1.18)	0.90 (0.53 to 1.27)	<.001	<.001
Kessler 6	N/A^e^	N/A	0.82 (0.53 to 1.11)	0.90 (0.56 to 1.23)	1.01 (0.64 to 1.38)	<.001	<.001
DERS-36^f^	N/A	N/A	0.83 (0.54 to 1.12)	0.98 (0.64 to 1.32)	1.18 (0.80 to 1.55)	<.001	<.001
MHC-SF^g^	N/A	N/A	−0.89 (−1.18 to −0.60)	−1.00 (−1.34 to −0.66)	−0.84 (−1.21 to −0.48)	<.001	<.001
R-LOT^h^	N/A	N/A	−0.72 (−1.01 to −0.43)	−0.90 (−1.24 to −0.56)	−0.79 (−1.16 to −0.43)	<.001	<.001
EQ-5D-3L Utility Index	N/A	N/A	−0.58 (−0.78 to −0.29)	−0.63 (−0.96 to −0.29)	−0.50 (−0.87 to −0.14)	<.001	<.001
EQ-5D-3L Health Rating	N/A	N/A	−0.58 (−0.87 to −0.29)	−0.34 (−0.67 to −0.01)	−0.56 (−0.92 to −0.19)	<.001	<.001

^a^*P* value was based on Mann-Kendall test.

^b^FDR: false recovery rate.

^c^GAD-7: Generalized Anxiety Disorder 7.

^d^PHQ-9: Patient Health Questionnaire 9.

^e^N/A: not applicable.

^f^DERS-36: Difficulties in Emotional Regulation 36.

^g^MHC-SF: Mental Health Continuum—Short Form.

^h^R-LOT: Revised Life Orientation Test.

### Preliminary Comorbidity and Prevention Intervention Outcome Effects

**Figure 3:**
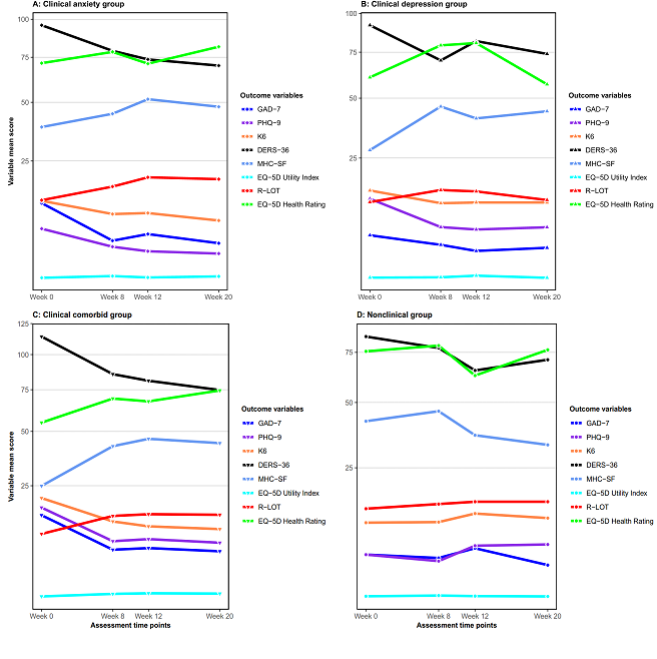
Intervention outcomes over time by preintervention diagnostic presentation subgroups. DERS-36: Difficulties in Emotional Regulation 36; GAD-7: Generalized Anxiety Disorder 7; K-6: Kessler 6; MHC-SF: Mental Health Continuum—Short Form; PHQ-9: Patient Health Questionnaire 9; R-LOT: Revised Life Orientation Test.

Figure 3, Tables 3 and 4, and Multimedia Appendix 7 display the results of the outcome measures across the scheduled assessment time points when categorizing the participants by their preintervention clinical diagnostic presentation (clinical anxiety only, clinical depression only, clinical comorbid anxiety and depression, and nonclinical anxiety and/or depression) to examine both the comorbidity and preventative intervention effects of Life Flex. The results indicated that even after adjusting for multiple tests, Life Flex produced significant changes for all measures for 2 subgroups, anxiety only (GAD-7, FDR<.001; PHQ-9, FDR<.001; K-6, FDR<.001; DERS-36, FDR=.004; MHC-SF, FDR=.01; R-LOT, FDR<.001; EQ-5D-3L Utility Index, FDR=.02; and EQ-5D-3L Health Rating, FDR=.04) and comorbid anxiety and depression (GAD-7, FDR<.001; PHQ-9, FDR<.001; K-6, FDR<.001; DERS-36, FDR<.001; MHC-SF, FDR<.001; R-LOT, FDR<.001; EQ-5D-3L Utility Index, FDR<.001; and EQ-5D-3L Health Rating, FDR<.001). The depression-only group showed significant changes in 4 measures, GAD-7 (FDR=.02), PHQ-9 (FDR<.002), K-6 (FDR=.02), and MHC-SF (FDR=.02). However, Life Flex did not result in preventative intervention effects for the nonclinical subgroup on any outcome measures. The treatment effect sizes for the clinical comorbid subgroup were large for every outcome measure at every time point, except for the EQ-5D-3L Health Rating, in which a medium effect size was observed at the postintervention and 1-month follow-up assessments.

**Table 3:** Descriptive of intervention outcomes at preintervention, postintervention, and follow-up scheduled assessment time points by preintervention clinical diagnostic presentation subgroups.

			Preintervention: week 0 (n=241)					Postintervention: week 8 (n=58-61)					1-month follow-up: week 12 (n=41)					3-month follow-up: week 20 (n=34-35)		
			Values, n (%)		Values, mean (SD)		Values, n (%)			Values, mean (SD)		Values, n (%)			Values, mean (SD)		Values, n (%)			Values, mean (SD)
**GAD-7^a^**																				
	Anxiety	26 (10.8)		12.35 (3.53)		9 (14.8)			4.78 (2.33)		7 (17.1)			5.86 (1.86)		5 (14.3)			4.40 (2.70)	
	Depression	23 (9.5)		5.43 (1.67)		6 (9.8)			4.00 (4.77)		5 (12.2)			3.20 (2.59)		5 (14.3)			3.60 (2.88)	
	Comorbid	136 (56.4)		15.03 (4.13)		34 (55.7)			6.56 (6.08)		24 (58.5)			6.88 (6.15)		19 (54.3)			6.26 (5.88)	
	Nonclinical	56 (23.2)		5.05 (1.86)		12 (19.7)			4.58 (2.43)		5 (12.2)			6.00 (3.54)		6 (17.1)			3.67 (3.72)	
**PHQ-9^b^**																				
	Anxiety	26 (10.8)		6.81 (2.25)		9 (15)			3.89 (3.52)		7 (17.1)			3.29 (2.50)		5 (14.7)			3.00 (2.35)	
	Depression	26 (10.8)		12.91 (3.26)		6 (10)			6.83 (8.73)		5 (12.2)			6.40 (6.54)		5 (14.7)			6.80 (6.98)	
	Comorbid	136 (56.4)		17.37 (4.94)		33 (55)			8.33 (7.43)		24 (58.5)			8.79 (7.37)		19 (55.9)			8.00 (6.76)	
	Nonclinical	56 (23.2)		5.05 (2.54)		12 (20)			4.17 (2.52)		5 (12.2)			6.40 (3.21)		5 (14.7)			6.60 (5.41)	
**K-6^c^**																				
	Anxiety	26 (10.8)		12.92 (2.87)		9 (15)			9.78 (1.56)		7 (17.1)			10.00 (0.82)		5 (14.7)			8.40 (1.67)	
	Depression	23 (9.5)		15.04 (3.57)		6 (10)			11.83 (6.62)		5 (12.2)			12.00 (5.29)		5 (14.7)			12.20 (7.82)	
	Comorbid	136 (56.4)		20.53 (4.73)		33 (55)			13.24 (6.49)		24 (58.5)			11.92 (5.77)		19 (55.9)			11.21 (4.89)	
	Nonclinical	56 (23.2)		10.63 (2.73)		12 (20)			10.75 (2.56)		5 (12.2)			12.60 (5.22)		5 (14.7)			11.60 (5.03)	
**DERS-36^d^**																				
	Anxiety	26 (10.8)		95.88 (19.37)		9 (15.3)			79.00 (21.43)		7 (17.1)			73.71 (20.34)		5 (14.7)			70.00 (23.77)	
	Depression	23 (9.5)		92.04 (20.76)		6 (10.2)			70.17 (36.68)		5 (12.2)			81.80 (22.58)		5 (14.7)			74.00 (28.30)	
	Comorbid	136 (56.4)		114.21 (21.49)		32 (54.2)			85.63 (33.74)		24 (58.5)			81.04 (33.44)		19 (55.9)			74.79 (29.51)	
	Nonclinical	56 (23.2)		83.93 (23.07)		12 (20.3)			77.42 (21.38)		5 (12.2)			65.40 (16.56)		5 (14.7)			71.00 (7.97)	
**MHC-SF^e^**																				
	Anxiety	26 (10.8)		38.42 (11.93)		9 (15)			44.44 (10.69)		7 (17.1)			51.57 (10.21)		5 (14.7)			47.80 (13.29)	
	Depression	23 (9.5)		27.83 (11.01)		6 (10)			46.00 (21.42)		5 (12.2)			40.60 (18.81)		5 (14.7)			43.80 (17.12)	
	Comorbid	136 (56.4)		24.93 (12.14)		33 (55)			42.36 (20.02)		24 (58.5)			46.08 (17.43)		19 (55.9)			43.89 (19.64)	
	Nonclinical	56 (23.2)		41.95 (12.22)		12 (20)			46.17 (10.99)		5 (12.2)			36.40 (20.07)		5 (14.7)			32.80 (7.79)	
**R-LOT^f^**																				
	Anxiety	26 (10.8)		13.08 (4.99)		9 (15.3)			16.78 (4.27)		7 (17.1)			19.57 (3.05)		5 (14.7)			19.00 (5.39)	
	Depression	23 (9.5)		12.09 (3.60)		6 (10.2)			15.17 (5.88)		5 (12.2)			14.80 (3.96)		5 (14.7)			12.60 (4.98)	
	Comorbid	136 (56.4)		10.01 (4.99)		32 (54.2)			14.81 (6.76)		24 (58.5)			15.33 (6.15)		19 (55.9)			15.16 (5.01)	
	Nonclinical	56 (23.2)		13.71 (4.63)		12 (20.3)			14.83 (4.61)		5 (12.2)			15.40 (5.03)		5 (14.7)			15.40 (3.91)	
**ED-5D-3L Utility Index**																				
	Anxiety	26 (10.8)		0.77 (0.11)		9 (15.5)			0.88 (0.11)		7 (17.1)			0.79 (0.19)		5 (14.7)			0.86 (0.08)	
	Depression	23 (9.5)		0.75 (0.11)		6 (10.3)			0.77 (0.23)		5 (12.2)			0.87 (0.13)		5 (14.7)			0.74 (0.31)	
	Comorbid	136 (56.4)		0.61 (0.19)		31 (53.4)			0.77 (0.23)		24 (58.5)			0.81 (0.17)		19 (55.9)			0.79 (0.19)	
	Nonclinical	56 (23.2)		0.86 (0.14)		12 (20.7)			0.90 (0.11)		5 (12.2)			0.87 (0.12)		5 (14.7)			0.85 (0.23)	
**ED-5D-3L Health Rating**																				
	Anxiety	26 (10.8)		71.46 (10.89)		9 (15.5)			78.33 (10.31)		7 (17.1)			71.14 (28.82)		5 (14.7)			81.60 (11.63)	
	Depression	23 (9.5)		60.70 (16.16)		6 (10.3)			79.17 (18.28)		5 (12.2)			80.60 (11.15)		5 (14.7)			57.00 (34.74)	
	Comorbid	136 (56.4)		54.86 (21.41)		31 (53.4)			69.29 (23.46)		24 (58.5)			67.50 (24.16)		19 (55.9)			74.53 (17.11)	
	Nonclinical	56 (23.2)		75.55 (12.69)		12 (20.7)			78.75 (14.60)		5 (12.2)			62.80 (34.41)		5 (14.7)			76.40 (5.46)	

^a^GAD-7: Generalized Anxiety Disorder 7.

^b^PHQ-9: Patient Health Questionnaire 9

^c^K-6: Kessler 6.

^d^DERS-36: Difficulties in Emotional Regulation 36.

^e^MHC-SF: Mental Health Continuum—Short Form.

^f^R-LOT: Revised Life Orientation Test.

**Table 4:** Change in intervention outcomes at all scheduled assessments over time by preintervention clinical diagnostic presentation subgroups.

		Week 0 vs 3, Cohen *d* (95% CI)	Week 0 vs 5, Cohen *d* (95% CI)	Week 0 vs 8, Cohen *d* (95% CI)	Week 0 vs 12, Cohen *d* (95% CI)	Week 0 vs 20, Cohen *d* (95% CI)	*P* value^a^	FDR^b^
**GAD-7^c^**								
	Anxiety	1.24 (0.35 to 2.13)	2.07 (0.95 to 1.46)	2.31 (1.36 to 3.23)	1.98 (1.00 to 2.94)	2.32 (1.78 to 3.43)	<.001	<.001
	Depression	0.04 (−0.86 to 0.94)	0.26 (−0.95 to 1.46)	0.56 (−0.35 to 1.47)	1.21 (0.18 to 2.22)	0.96 (−0.05 to 1.95)	.01	.02
	Comorbid	1.16 (0.78 to 1.53)	1.85 (1.39 to 2.30)	1.85 (1.42 to 2.27)	1.82 (1.34 to 2.30)	2.01 (1.47 to 2.53)	<.001	<.001
	Nonclinical	−0.07 (−0.65 to 0.52)	0.16 (−0.59 to 0.90)	0.24 (−0.39 to 0.86)	−0.47 (−1.39 to 0.45)	0.67 (−0.19 to 1.51)	.39	.48
**PHQ-9^d^**								
	Anxiety	1.15 (0.26 to 2.02)	0.81 (−0.18 to 1.78)	1.12 (0.31 to 1.91)	1.53 (0.61 to 2.44)	1.69 (0.62 to 2.72)	<.001	<.001
	Depression	1.31 (0.34 to 2.26)	0.99 (−0.26 to 2.21)	1.27 (0.30 to 2.22)	1.65 (0.57 to 2.70)	1.51 (0.44 to 2.54)	<.001	.002
	Comorbid	0.91 (0.54 to 1.28)	1.47 (1.03 to 1.90)	1.64 (1.22 to 2.06)	1.60 (1.13 to 2.07)	1.81 (1.28 to 2.33)	<.001	<.001
	Nonclinical	0.18 (−0.41 to 0.76)	−0.25 (−0.99 to 0.49)	0.35 (−0.28 to 0.97)	−0.52 (−1.44 to 0.40)	−0.55 (−1.46 to 0.38)	.85	.85
**K-6^e^**								
	Anxiety	N/A^f^	N/A	1.20 (0.38 to 2.01)	1.12 (0.26 to 2.00)	1.65 (0.59 to 2.69)	<.001	<.001
	Depression	N/A	N/A	0.75 (−0.18 to 1.66)	0.78 (−0.21 to 1.77)	0.63 (−0.36 to 1.61)	.01	.02
	Comorbid	N/A	N/A	1.42 (1.01 to 1.83)	1.76 (1.28 to 2.23)	1.96 (1.43 to 2.49)	<.001	<.001
	Nonclinical	N/A	N/A	−0.05 (−0.67 to 0.58)	−0.67 (−1.59 to 0.26)	−0.33 (−1.25 to 0.59)	.47	.54
**DERS-36^g^**								
	Anxiety	N/A	N/A	0.85 (0.06 to 1.63)	1.13 (0.24 to 2.01)	1.29 (0.27-2.29)	.002	.004
	Depression	N/A	N/A	0.95 (0.01 to 1.88)	0.49 (−0.49 to 1.46)	0.82 (−0.18 to 1.80)	.06	.09
	Comorbid	N/A	N/A	1.18 (0.77 to 1.58)	1.40 (0.94 to 1.86)	1.75 (1.22 to 2.26)	<.001	<.001
	Nonclinical	N/A	N/A	0.29 (−0.34 to 0.91)	0.82 (−0.11 to 1.74)	0.58 (−0.35 to 1.50)	.045	.07
**MHC-SF^h^**								
	Anxiety	N/A	N/A	−0.52 (−1.28 to 0.25)	−1.13 (−2.00 to −0.24)	−0.77 (−1.74 to 0.21)	.007	.01
	Depression	N/A	N/A	−1.34 (−2.30 to −0.36)	−1.02 (−2.02 to −0.01)	−1.31 (−2.33 to −0.27)	.014	.02
	Comorbid	N/A	N/A	−1.25 (−1.65 to −0.84)	−1.62 (−2.09 to −1.15)	−1.43 (−1.94 to −0.92)	<.001	<.001
	Nonclinical	N/A	N/A	−0.35 (−0.98 to 0.28)	0.43 (−0.49 to 1.35)	0.76 (−0.16 to 1.69)	.58	.62
**R-LOT^i^**								
	Anxiety	N/A	N/A	−0.77 (−1.54 to 0.02)	−1.39 (−2.28 to −0.48)	−1.17 (−2.17 to −0.16)	<.001	<.001
	Depression	N/A	N/A	−0.75 (−1.66 to 0.18)	−0.74 (−1.72 to 0.25)	−0.13 (−1.10 to 0.84)	.22	.28
	Comorbid	N/A	N/A	−0.90 (−1.29 to −0.50)	−1.03 (−1.48 to −0.58)	−1.03 (−1.52 to −0.54)	<.001	<.001
	Nonclinical	N/A	N/A	−0.24 (−0.87 to 0.38)	−0.36 (−1.27 to −0.56)	−0.38 (−1.28 to 0.55)	.42	.49
**EQ-5D-3L Utility Index**								
	Anxiety	N/A	N/A	−1.01 (−1.80 to −0.21)	−0.19 (−1.02 to 0.65)	−0.80 (−1.77 to 0.19)	.01	.02
	Depression	N/A	N/A	−0.12 (−1.02 to 0.78)	−1.03 (−2.03 to −0.01)	0.07 (−0.90 to 1.04)	.16	.21
	Comorbid	N/A	N/A	−0.80 (−1.20 to −0.40)	−1.04 (−1.49 to −0.59)	−0.92 (−1.41 to −0.42)	<.001	<.001
	Nonclinical	N/A	N/A	−0.28 (−0.90 to 0.35)	−0.07 (−0.98 to 0.85)	0.12 (−0.80 to 1.03)	.54	.60
**EQ-5D-3L Health Rating**								
	Anxiety	N/A	N/A	−0.64 (−1.41 to 0.14)	0.02 (−0.81 to 0.85)	−0.92 (−1.90 to 0.07)	.02	.04
	Depression	N/A	N/A	−1.11 (−2.05 to −0.16)	−1.28 (−2.30 to −0.25)	0.18 (−0.79 to 1.15)	.04	.05
	Comorbid	N/A	N/A	−0.66 (−1.06 to −0.26)	−0.58 (−1.02 to −0.14)	−0.94 (−1.43 to −0.45)	<.001	<.001
	Nonclinical	N/A	N/A	−0.25 (−0.87 to 0.38)	0.84 (−0.09 to 1.76)	−0.07 (−0.98 to 0.85)	.68	.70

^a^*P* value was based on Mann-Kendall test.

^b^FDR: false recovery rate.

^c^GAD-7: Generalized Anxiety Disorder 7.

^d^PHQ-9: Patient Health Questionnaire 9.

^e^K-6: Kessler 6.

^f^N/A: not applicable.

^g^DERS-36: Difficulties in Emotional Regulation 36.

^h^MHC-SF: Mental Health Continuum—Short Form.

^i^R-LOT: Revised Life Orientation Test.

### Program Use

Of the 241 participants, 219 (90.9%) logged in at least once and viewed at least some of the Introduction module; 161 (66.8%) completed the Introduction module; 91 (37.8%) completed module 1 (Increasing Biological Flexibility); 60 (24.9%) completed module 2 (Increasing Emotional Flexibility); 37 (15.4%) completed module 3 (Increasing Thinking Flexibility); 31 (12.9%) completed module 4 (Increasing Behavioral Flexibility); 22 (9.1%) completed module 5 (Increasing Wellness Flexibility); and 13 (5.4%) completed module 6 (Increasing Life Flexibility).

### Postintervention Acceptability and Qualitative Intervention Impressions

#### Overview

Participants were asked to rate the program using 8 multiple-choice questions and to discuss the best and worst parts of the Life Flex program after the intervention.

#### Posttreatment Acceptability and Satisfaction (TAS-Q) Ratings (n=51)

When asked to rate the *quality* of the Life Flex program, 88% (45/51) of the participants rated it as good to excellent; when asked whether they received the *right kind of information* and *strategies*, 94% (48/51) of the participants said yes; when asked to what extent Life Flex *met their needs*, 90% (46/51) of the participants said “some” to “all” of their needs were met; when asked if they would *recommend* Life Flex to a friend, 96% (49/51) of the participants said yes; when asked about how satisfied they were with the *amount of information* they received, 90% (46/51) of the participants said they were satisfied to very satisfied; when asked about how much the Life Flex program had helped them to *effectively deal with their problems*, 88% (45/51) of the participants said that it helped them; when asked overall, in a *general sense, how satisfied* they were with the Life Flex program, 80% (41/51) of the participants said they were satisfied to highly satisfied; and when asked if they would *return to Life Flex* into the future if required, 88% (45/51) of the participants said yes.

#### Posttreatment Qualitative Life Flex Intervention Responses (n=51)

When participants were asked about the *best parts* of the Life Flex intervention, common responses included great information and strategies, activities, videos explaining things, choice in strategies, holistic focus, brain plasticity information, the inclusion of the biological explanations of depression and anxiety, guidance to help them move forward, ability to self-pace, mood monitoring, the quality of the information (well-explained and comprehensive), learning more about emotions, genuine caring expressed in content, and the variety of presentation formations. When asked about the *worst parts* of the Life Flex intervention, common responses included not enough reminders, need for a better self-monitoring tool, no access to a hard copy version of the Life Flex intervention, content being somewhat wordy at times, limited intervention feedback on how they were going, no accessibility without an internet connection, and the lack of mobile app version.

#### Exploration of Qualitative Impressions of the Transdiagnostic Intervention Content Expansion (n=81)

We explored the participants’ impressions of the first Life Flex module (the Introduction module), which provides them with an overview and summary of the entire program (Multimedia Appendix 1) and an introduction to the key theories (eg, CBT and positive psychology) and concepts (eg, brain plasticity and allostasis; including video explainers). In the following paragraph, we present the results of the participants’ review of this module (ie, at the beginning of each module, participants were able to optionally undertake a brief review of the previous module. This provided an opportunity for both the participant and the study researchers to receive feedback on the previous module).

For the review of the Introduction module, we received 98 entries, with 17 (17%) duplicates (leaving n=81, 83%, entries from different participants). When asked what participants found most useful within the Introduction module, we observed that many of the entries were general nonspecific comments (ie, described participants’ general impressions such as “a good introduction/summary/overview, clear/informative” of the intervention). However, when looking at specific responses, the most common themes were as follows: (1) the introduction to the brain plasticity and biology of stress response information and videos (23 specific comments); (2) motivational interviewing–related activities (eg, pros and cons activity, things I would like to change, commitment plan; 15 specific comments) and (3) the recommended positive affect–focused offline activity (successful events or positive self-statements; 13 specific comments). Textbox 1 provides examples of some of the comments.

**Representative specific qualitative statements made about the useful aspects of the Introduction module.**

**Representative qualitative statements by theme**

- Brain plasticity and stress response
  - “The realisation that I can change my brain and that I need not be a victim of my ill health and mood. That i (sic) am capable.”
  - “The information (or reiteration) of neural plasticity and that it’s possible to change the way one’s brain function and there is no need to feel stuck with the status quo.”
  - “The simple explanation of the possibility of changing the way the brain processes.”
  - “I found it useful to read about the flexibility & plasticity of the brain and that through using certain strategies I can change my negative thought loop.”
  - “Helpful to reinforce the notion that things can change and that the brain can be rewired positively.”
  - “It made me aware of aspects of my condition I did not know—how we are physically and mentally conditioned to react the way we do, and can change that response.”
  - “Gave a good indication of what to expect in the program. The emphasis on the notion that change comes from changing neural pathways and that the exercises in the program are designed to help effect that change, is positive and motivating.”
  - “1. The discussion of primitive, middle and frontal brain relationships. 2. The concepts of sympathetic and parasympathetic nervous systems.”
- Motivational interviewing activities
  - “The opportunity to write things down and commit to a plan. The opportunity to revisit those thoughts.”
  - “The pros and cons section was also very helpful—it was really important for me to acknowledge to myself my fear of failure.”
  - “I very much liked the information provided as it was clear and yet simple. I also liked the idea of a contract and the setting of parameters to help me follow through on the program.”
  - “Knowing change its (sic) possible and how to initiate the change. The introduction made me excited to continue.”
  - [W]riting (sic) down what I wanted to get from this and why.”
  - “All of it was, writing down what I wanted to achieve from the course.”
- Recommended offline activities (positive affect focus)
  - “I liked the selection of your encouragement (positive event or positive statement) to focus on. It might sounds (sic) silly but having the choice and selecting something for you made me feel a lot more in control of/involved in the process.”
  - “The affirmation—'I can change my brain’ has really stuck with me.”
  - “[W]riting [sic] down the event that I felt good about, and remembering it several times a day was extremely powerful. I felt good about myself, and stopped the negative thoughts that I was having as well.”
  - “Positive affirmations led to me standing up for myself a bit more.”
  - “It explained the programme and how it is believed that changes to thought patterns can occur. It provided an opportunity for reflection and a tool of focusing on a positive statement which I have been using when my brain gets too busy.”
  - “Generating 3 things to say to self at intervals throughout day = seems like a good thing to do.”

## Discussion

### Principal Findings

The main aim of this study was to explore the potential effectiveness and acceptability feasibility of Life Flex, a fully automated self-help biopsychosocial transdiagnostic digital health intervention designed to decrease anxiety and/or depression. Significant reductions were found in both primary outcome measures (anxiety and depression) from preintervention assessment through to the 3-month follow-up. The secondary outcomes also showed significant improvements, including reduced psychological distress and improved emotion regulation; emotional, social, and psychological well-being; optimism; and health-related quality of life. This overall improvement in the primary outcomes in this study is consistent with those seen in other transdiagnostic digital programs with therapist assistance. For example, in a meta-analysis by Newby et al [21], therapist-assisted transdiagnostic digital treatments for anxiety and/or depression yielded uncontrolled effect sizes of 0.96. Life Flex’s within-group treatment effect sizes at the postintervention time point and 1- and 3-month follow-ups ranged between Cohen *d*=0.96 and Cohen *d*=1.33 for anxiety and Cohen *d*=0.84 and Cohen *d*=0.93 for depression. Although a direct comparison is impossible, Batterham et al [22] also reported significant between-group changes in the PHQ-9 and other anxiety-related measures (panic and social anxiety) using their self-help transdiagnostic digital program, relative to an attentional control condition, and the results are similar to our results (albeit within-group changes in depression and anxiety). Overall, it is encouraging to observe such similar effect size changes in our digital intervention relative to digital plus therapist-supported transdiagnostic interventions (or even face-to-face), and this provide early support that for some people, a fully automated self-help transdiagnostic digital approach to address anxiety and/or depression can be possible.

The second aim was to group participants based on their preintervention clinical diagnostic presentation and to evaluate possible comorbidities and preventative intervention effects. When evaluating the diagnostic subgroupings against the primary and secondary outcome measures, those with clinical comorbid presentations appeared to improve the most, whereas those with nonclinical subthreshold presentations improved the least. The clinical comorbidity intervention effect sizes obtained in this study at the postintervention time point or follow-up were larger (between Cohen *d*=1.82 and Cohen *d*=2.01 for anxiety and between Cohen *d*=1.60 and Cohen *d*=1.81 for depression for the comorbidity subgroup), relative to the analysis results for the whole group (between Cohen *d*=0.96 and Cohen *d*=1.33 for anxiety and between Cohen *d*=0.84 and Cohen *d*=0.93 for depression).

However, in terms of preventative intervention effects, no significant changes were found in the primary or secondary outcome measures in the nonclinical subgroup. Therefore, unlike the adolescent transdiagnostic digital intervention study results obtained by Schmitt et al [25], Life Flex did not produce preventative intervention effects for anxiety and depression. In terms of core differences between the 2 studies, the study by Schmitt et al [25] included therapist assistance (and researcher contact), and their intervention was tested on adolescents. Overall, the preliminary results suggest that Life Flex has good clinical comorbidity intervention outcome effects but no preventative outcome intervention effects. Further investigation is therefore required using longer follow-up periods and the inclusion of a control condition to best test for any possible preventative intervention effects, as well as to serve as a replication study for the comorbidity intervention effects.

The third and final aim of the study was to measure postintervention program acceptability, including participant impressions of the content expansion, using both quantitative and qualitative responses. The results suggest that Life Flex was well received by participants. In general, participants found the program quality to be high, and their satisfaction with the program meant that they were also willing to recommend it to a friend (96.1%) and return to it in the future if required (88.3%). However, some of the qualitative responses about the worst parts of the program suggested room for improvement (eg, adding more reminders, developing a more useful self-monitoring tool, decreasing wordiness, and increasing program progress information). In relation to transdiagnostic program content expansion and based on qualitative responses (review of Introduction module and postintervention TAS-Q data), it would appear that the biologically based concepts and positive affect and well-being content, as well as the addition (and choice) of the increasing biological and wellness flexibility strategies, were welcomed, beneficial, and enjoyable additions. However, whether these additions contributed to significant intervention outcome changes is unknown and will require formal testing in the future.

Although UP and CBT are effective transdiagnostic treatments for emotional disorders, they are driven by a psychological framework that focuses largely on emotion, cognition, and behavior. Nonpharmacological biologically focused interventions (ie, lifestyle interventions that work on enabling brain plasticity and decreasing allostatic load on the body, such as increasing physical activity and brain and gut nutrition) that improve emotional regulation [55] and increase positive emotions [56] do show promise as useful additions based on the general results of this study. Therefore, more dismantling research on transdiagnostic intervention content and their associated mechanisms of change is required to hopefully arrive at the most time-efficient, effective, and adherent self-help transdiagnostic digital intervention protocol possible. In addition, future studies might also consider collecting blood biomarkers to test for potential biological changes alongside psychological changes. For example, increasing physical activity has been shown to modulate several core biomarkers of neuroprogression, including neurotrophins and oxidative stress [36,57], as well as decrease self-reported symptoms of depression [58] and anxiety [36].

### Limitations

Although this was a feasibility trial, a limitation of this study was the absence of a control condition, which makes it impossible to conclude whether the results of the Life Flex intervention were due to time passing, the use of other forms of support, or nonspecific effects. In addition, as with any intervention research, this study suffers from self-selection bias, limiting the generalizability of its findings. Moreover, as this study tested a fully automated self-help digital intervention, conducting clinical assessments to confirm the diagnosis would have voided the real-world, self-help elements of the study. However, the diagnosis relied on validated diagnostic self-report measures (GAD-7 and PHQ-9), which can be subjective. Furthermore, a longer follow-up period would have assisted in better measuring the longer-term maintenance and possible preventative intervention effects. Another limitation was the low scheduled assessment completion rate following the preintervention assessment. Although this is not uncommon for fully automated self-help digital health intervention trials [59], correction to the data set is typically required, such as multiple imputation. However, given the higher-than-expected amount of missing data in this feasibility trial, we used the completer analysis data to demonstrate potential effectiveness and acceptability feasibility. Our comparison of the observed demographics between completer and missing data demonstrated no significant difference, which suggests that the data would be missing at random, providing greater confidence in the results reported. However, caution (and further replication) must be exercised when considering these preliminary results.

### Conclusions

In summary, the results of this trial are promising, despite the absence of a control condition, self-selection bias, and reliance on self-report measures for diagnosis, as this study represents real-world conditions that provide people with direct access to their intervention of choice. Given the potential rise of mental health issues associated with more recent global events (eg, pandemic and economic downturn), the introduction of this scalable, fully automated self-help biopsychosocial transdiagnostic digital intervention is timely and could have widespread benefits. Although the exploratory results found in this trial are encouraging, studies that include comparator conditions are required. Importantly, 2 subsequent Life Flex studies have recently been completed (with comparator randomized conditions) and are currently being written up or have recently been accepted by the *Journal of Medical Internet Research* [60].

In addition, since completing this exploratory feasibility trial, we have updated the Life Flex program (now housed within the HealthZone digital platform) to address qualitative feedback received from this study (eg, a new interactive self-monitoring tool with wearable data integration, which includes several *just-in-time* intervention algorithms, more reminders, visual summary progress feedback, and the editing of some content to decrease wordiness). Furthermore, given the role that motivation and self-efficacy plays in engagement and behavior change, especially in the context of fully automated self-help digital programs, in one of our current trials, we have added a digital conversational (chat) agent to increase engagement by supporting those who experience difficulties with motivation and/or self-efficacy while undertaking the program. The conversational agent was based on the transtheoretical model of behavior change and incorporated motivational interviewing and positive psychological techniques. It is hoped that the introduction of this digital agent will also act as a pseudoform of social support, representing another step toward a more engaging self-help digital program.

In conclusion, given the convenience and availability of digital interventions, this fully automated self-help transdiagnostic intervention is a potential way to address the accessibility of treatment options for anxiety and/or depression. However, further investigation using large-scale randomized controlled trials is required.

## Appendix

Multimedia Appendix 1 Description of Life Flex digital health introduction and core modules.

Multimedia Appendix 2 Missing data pattern of key variables across time.

Multimedia Appendix 3 Distribution of the sociodemographic and clinical characteristics comparing participants who completed all scheduled assessments (complete data set) versus participants who did not (missing data set).

Multimedia Appendix 4 Descriptive of outcomes at preintervention by clinical diagnostic presentation subgroup.

Multimedia Appendix 5 Sociodemographic and clinical characteristics of the sample at preintervention by clinical diagnostic presentation subgroup.

Multimedia Appendix 6 Study characteristics at preintervention (N=241).

Multimedia Appendix 7 Descriptive of intervention outcomes at the scheduled during intervention assessment time point by preintervention clinical diagnostic presentation subgroup.

## Abbreviations

AD: anxiety disorder
CBT: cognitive behavioral therapy
DERS-36: Difficulties in Emotional Regulation 36
FDR: false discovery rate
GAD-7: Generalized Anxiety Disorder 7
K-6: Kessler 6
MHC-SF: Mental Health Continuum—Short Form
PHQ-9: Patient Health Questionnaire 9
R-LOT: Revised Life Orientation Test
TAS-Q: Treatment Acceptability and Satisfaction Questionnaire
UP: Unified Treatment Protocol

## Data Availability

The raw, nonidentifiable data supporting the conclusions of this paper will be made available by the corresponding author, following a request to Federation University Human Research Ethics Committee for access to these data (as per the study’s approved ethical application).
