# Supplementary material for: A Fully Automated Self-help Biopsychosocial Transdiagnostic Digital Intervention to Reduce Anxiety and/or Depression and Improve Emotional Regulation and Well-being: Pre–Follow-up Single-Arm Feasibility Trial
Source: JMIR Form Res. 2023 May 30;7:e43385. doi: 10.2196/43385 (PMC10265433; doi:10.2196/43385)
Supplement: Multimedia Appendix 1 [file formative_v7i1e43385_app1.docx]

**Multimedia Appendix 1.**

Description of Life Flex digital health introduction and core modules.

| Module | Description |
| --- | --- |
| Introduction | This module provides an overview of the program; the key theories driving the program and the core concepts used within the program (eg, CBT^a^, emotional regulation, allostasis and allostatic load, HRV^b^, and positive affect); a discussion around brain plasticity and 3 brain plasticity explainer videos; psychoeducation about stress, anxiety, depression, and use of motivational interviewing techniques to promote motivation toward change, overcoming barriers, and commitment to undertaking the program. The module concludes with a recommended offline skill-building activity and an additional resource. Here, the offline activity was to start self-monitoring anxiety and depressive symptoms and practicing either the “Successful Events” or “Positive Self-statements” activity. The additional resource was for suicidal thoughts and keeping safe (safety plan). There are 2 other videos in this module (an Introduction module summary video and an explainer video on why we put Life Flex together). |
| Increasing biological flexibility | This module provides a review activity of the previous module; a rationale for the current module; discusses the biology of the stress response (activation and deactivation [allostasis] of the stress response, allostatic load, the role of autonomic system functioning, and HRV); information on understanding biological and psychological interactions; what “biological flexibility” is and what techniques can be used to increase biological flexibility (eg, increasing physical activity, brain and gut nutrition, breathing control, brain training, mindfulness, progressive muscle relaxation, and visual imagery); and the associated tools (eg, instructions, guided audios, videos, PDF downloads, games, digital tracking, and monitoring forms and activities) to practice one or more of the strategies. The offline activity recommendation was to undertake one of the strategies. The additional resources provided were information on sleep hygiene, alcohol and drug use, and goal-setting. In addition to the videos about the strategies, there was a module summary video and a video explainer on the biology of the stress response. |

| Increasing emotional flexibility (split as parts A and B minimodules) | Part A provides a review activity of the previous module; a rationale for the current module; overview of what emotions are, increasing emotional awareness; and understanding “emotional flexibility,” techniques to improve emotional flexibility (eg, adopting an objective stance, focusing awareness to the present moment, allowing emotions to take their natural course, and riding the emotional wave). Part B focused on understanding what emotion regulation is, sequence of emotional regulation events, and different emotional regulation strategy types. There was an experiential video-based activity to compare and contrast “response modulation” emotional regulation strategies of suppression, cognitive appraisal, and acceptance to help participants develop knowledge about what each type is and when it might be most appropriate to use it. This module concluded with discussing “things not in their control” and assisting them with decision-making about when it might be better for them to change, accept, or let go when each new stressful situation occurs. Participants are also introduced to a “Loving Kindness” meditation activity. In this module, there were 5 explainer videos (introduction summary, loving kindness instruction, and 3 experiential emotional regulation strategy practice videos). Offline activity recommendations included monitoring emotions and emotional regulation strategy use and loving kindness mediation practice. The additional resource was on distress tolerance. |
| --- | --- |
| Increasing thinking flexibility | This module provides a review activity of the previous module; a rationale for the current module; and looked at what thoughts are and their role, core beliefs and unhelpful thinking styles, automatic thoughts, and what “thinking flexibility” is (eg, identifying automatic thoughts and thought challenging). This module included 2 videos (module summary and thought challenging technique summary), and participants were reminded about change, acceptance, or letting go decision-making (introduced in the previous module). The module concludes with a brief discussion about worry and rumination and how to reduce it. Participants were asked to monitor their automatic thoughts, examine the evidence for their thoughts, and formulate more helpful thoughts. The additional resources included information on problem-solving (using the SMART^c^ technique) and on different cognitive biases. |

| Increasing behavioral flexibility | This module provides a review activity of the previous module, a rationale for the current module, looked at the influence of what we do on how we think and feel and vice versa, general coping methods, anxiety and avoidance, depression and low arousal, and how to increase “behavioral flexibility” through reducing avoidance through gradual exposure (for anxiety) and increasing activity through behavioral activation (for depression). There are 3 videos in this module (module summary, summary of exposure therapy steps, and a summary of the behavioral activation steps). Offline activities involved commencing with either the exposure or behavioral activation strategy (depending on which of the 2 was their main current concern). The additional resource was information on different communication styles. |
| --- | --- |
| Increasing wellness flexibility | This module provides a review activity of the previous module and a rationale for the current module, and it discusses positive and negative affect/emotions and wellness and how to increase “wellness flexibility” through various strategies (eg, gratitude, acts of kindness, personal strengths building, openness to experience, compassionate meditation, and cognitive bias modification) and a range of tools (eg, instructions, guided audios, videos, PDF downloads, games, digital tracking, and monitoring forms and activities) to practise one or more of the strategies. The additional resource in this module was about social connectedness. There was 1 video in this module (module summary), alongside the various videos within the wellness flexibility strategies instructions. |
| Increasing life flexibility | This module provides a review activity of the previous module, a recap of the Life Flex program by integrating all the previous teachings into a summary, as well as a reminder of what each previous module covered (and all 5 summary module videos), an opportunity to reflect on progress with feedback, and how to stay on track (standard relapse prevention activities). Participants were asked to continue with their daily monitoring, a wellness flexibility strategy, and exposure or behavioral activation tasks. After the program, there was a 2-page “booster” module (provided after 3 weeks) inviting them to undertake a 30-day challenge (choosing a new increasing biological or wellness flexibility strategy, starting a new hobby or revisit thought challenging, and undergoing exposure or behavioral activation if they were still having difficulties in these areas) and to say goodbye. |

^a^CBT: cognitive behavior therapy.

^b^HRV: heart rate variability.

^c^SMART: Specific, Measurable, Attainable, Realistic, Timely
