# Supplementary material for: A Fully Automated Self-help Biopsychosocial Transdiagnostic Digital Intervention to Reduce Anxiety and/or Depression and Improve Emotional Regulation and Well-being: Pre–Follow-up Single-Arm Feasibility Trial
Source: JMIR Form Res. 2023 May 30;7:e43385. doi: 10.2196/43385 (PMC10265433; doi:10.2196/43385)
Supplement: Multimedia Appendix 2 [file formative_v7i1e43385_app2.doc]

**Multimedia Appendix 2.**

Missing data pattern of key variables across time

|  | Missing (number) | Total sample | Missing (%) |
| --- | --- | --- | --- |
| GAD-7 (week 0) | 0 | 241 | 0 |
| GAD-7 (week 3) | 175 | 241 | 72.61 |
| GAD-7 (week 5) | 197 | 241 | 81.74 |
| GAD-7 (week 8) | 180 | 241 | 74.69 |
| GAD-7 (week 12) | 200 | 241 | 82.99 |
| GAD-7 (week 20) | 206 | 241 | 85.48 |
| PHQ-9 (week 0) | 0 | 241 | 0 |
| PHQ-9 (week 3) | 175 | 241 | 72.61 |
| PHQ-9 (week 5) | 197 | 241 | 81.74 |
| PHQ-9 (week 8) | 181 | 241 | 75.1 |
| PHQ-9 (week 12) | 200 | 241 | 82.99 |
| PHQ-9 (week 20) | 207 | 241 | 85.89 |
| K-6 (week 0) | 0 | 241 | 0 |
| K-6 (week 8) | 181 | 241 | 75.1 |
| K-6 (week 12) | 200 | 241 | 82.99 |
| K-6 (week 20) | 207 | 241 | 85.89 |
| DERS-36 (week 0) | 0 | 241 | 0 |
| DERS-36 (week 8) | 182 | 241 | 75.52 |
| DERS-36 (week 12) | 200 | 241 | 82.99 |
| DERS-36 (week 20) | 207 | 241 | 85.89 |
| MHC-SF (week 0) | 0 | 241 | 0 |
| MHC-SF (week 8) | 181 | 241 | 75.1 |
| MHC-SF (week 12) | 200 | 241 | 82.99 |
| MHC-SF (week 20) | 207 | 241 | 85.89 |
| R-LOT (week 0) | 0 | 241 | 0 |
| R-LOT (week 8) | 182 | 241 | 75.52 |
| R-LOT (week 12) | 200 | 241 | 82.99 |
| R-LOT (week 20) | 207 | 241 | 85.89 |
| EQ-5D-3L Utility Index (week 0) | 0 | 241 | 0 |
| EQ-5D-3L Utility Index (week 8) | 183 | 241 | 75.93 |
| EQ-5D-3L Utility Index (week 12) | 200 | 241 | 82.99 |
| EQ-5D-3L Utility Index (week 20) | 207 | 241 | 85.89 |
| EQ-5D-3L Health Rating (week 0) | 0 | 241 | 0 |
| EQ-5D-3L Health Rating (week 8) | 183 | 241 | 75.93 |
| EQ-5D-3L Health Rating (week 12) | 200 | 241 | 82.99 |
| EQ-5D-3L Health Rating (week 20) | 207 | 241 | 85.89 |

Note: GAD-7 = Generalized Anxiety Disorder 7; PHQ-9 = Patient Health Questionnaire 9; K-6 = Kessler 6; DERS-36 = Difficulties in Emotional Regulation 36; MHC-SF = Mental Health Continuum—Short Form; R-LOT: Revised Life Orientation Test.
