# Supplementary material for: A Fully Automated Self-help Biopsychosocial Transdiagnostic Digital Intervention to Reduce Anxiety and/or Depression and Improve Emotional Regulation and Well-being: Pre–Follow-up Single-Arm Feasibility Trial
Source: JMIR Form Res. 2023 May 30;7:e43385. doi: 10.2196/43385 (PMC10265433; doi:10.2196/43385)
Supplement: Multimedia Appendix 3 [file formative_v7i1e43385_app3.doc]

**Multimedia Appendix 3**

Distribution of the sociodemographic and clinical characteristics comparing participants who completed all scheduled assessments (complete data set) versus participants who did not (missing data set).

| **Variables** | **Complete data^a^**  **(N = 35)** | **Missing data^a^**  **(N = 206)** | ***p*-value** | **FDR** |
| --- | --- | --- | --- | --- |
| **Gender identity** |  |  | .16 | .78 |
| Male | 16 (45.7%) | 69 (33.5%) |  |  |
| Female | 19 (54.3%) | 137 (66.5%) |  |  |
| **Country of birth** |  |  | .23 | .78 |
| Australia | 5 (14.3%) | 48 (23.3%) |  |  |
| Another country | 30 (85.7%) | 158 (76.7%) |  |  |
| **Country of residence** |  |  | .62 | .90 |
| Australia | 33 (94.3%) | 198 (96.1%) |  |  |
| Another country | 2 (5.7%) | 8 (3.9%) |  |  |
| **Aboriginal and Torres Strait Islander** |  |  | .04 | .54 |
| Neither Aboriginal nor Torres Strait Islander | 33 (94.3%) | 204 (99.0%) |  |  |
| Aboriginal or Torres Strait Islander | 2 (5.7%) | 2 (1.0%) |  |  |
| **Sexual orientation** |  |  | .28 | .79 |
| Heterosexual | 27 (77.1%) | 174 (84.5%) |  |  |
| Gay/Lesbian/Bisexual/Another/Rather not say | 8 (22.9%) | 32 (15.5%) |  |  |
| **Relationship status** |  |  | .82 | .90 |
| Single | 7 (20.0%) | 47 (22.8%) |  |  |
| Married | 16 (45.7%) | 97 (47.1%) |  |  |
| De-facto | 8 (22.9%) | 34 (16.5%) |  |  |
| Divorced/Separated/Widowed | 4 (11.4%) | 28 (13.6%) |  |  |
| **Education level** |  |  | .51 | .90 |
| Secondary | 8 (22.9%) | 28 (13.6%) |  |  |
| Vocational/TAFE | 8 (22.9%) | 44 (21.4%) |  |  |
| BA | 13 (37.1%) | 92 (44.7%) |  |  |
| Postgraduate | 6 (17.1%) | 42 (20.4%) |  |  |

| **Employment status** |  |  | .37 | .90 |
| --- | --- | --- | --- | --- |
| Full-time | 14 (40.0%) | 89 (43.2%) |  |  |
| Part-time | 7 (20.0%) | 34 (16.5%) |  |  |
| Volunteer | 4 (11.4%) | 21 (10.2%) |  |  |
| Studying | 1 (2.9%) | 22 (10.7%) |  |  |
| Home duties/carer | 2 (5.7%) | 15 (7.3%) |  |  |
| Disability support | 1 (2.9%) | 8 (3.9%) |  |  |
| Retired | 1 (2.9%) | 8 (3.9%) |  |  |
| Another status | 5 (14.3%) | 9 (4.4%) |  |  |
| **Annual income** |  |  | .74 | .90 |
| <$40,000 | 9 (27.3%) | 57 (29.8%) |  |  |
| $40,000-<$80,000 | 11 (33.3%) | 77 (40.3%) |  |  |
| $80,000-<$120,000 | 9 (27.3%) | 39 (20.4%) |  |  |
| ≥$120,000 | 4 (12.1%) | 18 (9.4%) |  |  |
| **Residential location** |  |  | .77 | .90 |
| City/Metropolitan | 23 (65.7%) | 130 (63.1%) |  |  |
| Rural/Remote/Regional | 12 (34.3%) | 76 (36.9%) |  |  |
| **Accessed physical health services in last 4 weeks** |  |  | .07 | .54 |
| No | 8 (22.9%) | 80 (38.8%) |  |  |
| Yes | 27 (77.1%) | 126 (61.2%) |  |  |
| **Accessed mental health services in last 4 weeks** |  |  | .65 | .90 |
| No | 22 (62.9%) | 121 (58.7%) |  |  |
| Yes | 13 (37.1%) | 85 (41.3%) |  |  |
| **Drink alcohol** |  |  | .07 | .54 |
| Never | 3 (8.6%) | 35 (17.0%) |  |  |
| Monthly or less | 12 (34.3%) | 31 (15.0%) |  |  |
| 2 - 4 times a month | 5 (14.3%) | 35 (17.0%) |  |  |
| 2 - 3 times a week | 10 (28.6%) | 57 (27.7%) |  |  |
| 4 or more times a week | 5 (14.3%) | 48 (23.3%) |  |  |

| **Use illicit drugs** |  |  | .24 | .78 |
| --- | --- | --- | --- | --- |
| Never | 19 (54.3%) | 144 (69.9%) |  |  |
| I used too, but not in the last 12 months | 12 (34.3%) | 39 (18.9%) |  |  |
| No more than 12 times in the last 12 months | 1 (2.9%) | 11 (5.3%) |  |  |
| 2 - 4 times a month | 1 (2.9%) | 7 (3.4%) |  |  |
| 2 - 3 times a week | 0 (0.0%) | 1 (0.5%) |  |  |
| 4 or more times a week | 2 (5.7%) | 4 (1.9%) |  |  |
| **Smoke cigarettes** |  |  | .20 | .78 |
| Never have | 12 (34.3%) | 107 (51.9%) |  |  |
| I used too, but not anymore | 16 (45.7%) | 63 (30.6%) |  |  |
| Not regularly, but once in a while | 1 (2.9%) | 10 (4.9%) |  |  |
| Yes | 6 (17.1%) | 26 (12.6%) |  |  |
| **Using psychotropic medication** |  |  | .68 | .90 |
| No | 10 (28.6%) | 66 (32.0%) |  |  |
| Yes | 25 (71.4%) | 140 (68.0%) |  |  |
| **Do you feel you have enough social support / meaningful connections with other people** |  |  | .87 | .90 |
| Not at all | 3 (8.6%) | 17 (8.3%) |  |  |
| A little | 9 (25.7%) | 51 (24.8%) |  |  |
| Somewhat | 12 (34.3%) | 66 (32.0%) |  |  |
| Much | 5 (14.3%) | 45 (21.8%) |  |  |
| Very much | 6 (17.1%) | 27 (13.1%) |  |  |
| **Anxiety** |  |  | .85 | .90 |
| GAD-7 ≥8 | 24 (68.6%) | 138 (67.0%) |  |  |
| GAD-7 <8 | 11 (31.4%) | 68 (33.0%) |  |  |
| **Depression** |  |  | .69 | .90 |
| PHQ-9 ≥10 | 24 (68.6%) | 134 (65.0%) |  |  |
| PHQ-9 <10 | 11 (31.4%) | 72 (35.0%) |  |  |

| **Current Panic Disorder symptoms** |  |  | .51 | .90 |
| --- | --- | --- | --- | --- |
| Never | 10 (28.6%) | 65 (31.6%) |  |  |
| Not now, but I used too | 8 (22.9%) | 27 (13.1%) |  |  |
| Sometimes | 10 (28.6%) | 64 (31.1%) |  |  |
| Yes | 7 (20.0%) | 50 (24.3%) |  |  |
| **Current Social Anxiety Disorder symptoms** |  |  | .73 | .90 |
| Never | 5 (14.3%) | 30 (14.6%) |  |  |
| Not now, but I used too | 2 (5.7%) | 19 (9.2%) |  |  |
| Sometimes | 13 (37.1%) | 87 (42.2%) |  |  |
| Yes | 15 (42.9%) | 70 (34.0%) |  |  |
| **Current Specific Phobia symptoms** |  |  | .96 | .96 |
| Never | 13 (37.1%) | 73 (35.4%) |  |  |
| Not now, but I used too | 4 (11.4%) | 19 (9.2%) |  |  |
| Sometimes | 9 (25.7%) | 60 (29.1%) |  |  |
| Yes | 9 (25.7%) | 54 (26.2%) |  |  |
| **Current Post Traumatic Stress Disorder symptoms** |  |  | .86 | .90 |
| Never | 8 (22.9%) | 61 (29.6%) |  |  |
| Not now, but I used too | 7 (20.0%) | 35 (17.0%) |  |  |
| Sometimes | 9 (25.7%) | 47 (22.8%) |  |  |
| Yes | 11 (31.4%) | 63 (30.6%) |  |  |
| **Current Obsessive-Compulsive Disorder symptoms** |  |  | .67 | .90 |
| Never | 9 (25.7%) | 41 (19.9%) |  |  |
| Not now, but I used too | 2 (5.7%) | 24 (11.7%) |  |  |
| Sometimes | 11 (31.4%) | 69 (33.5%) |  |  |
| Yes | 13 (37.1%) | 72 (35.0%) |  |  |
| **Severity of falling asleep** |  |  | .14 | .78 |
| None | 14 (40.0%) | 57 (27.7%) |  |  |
| Mild to very severe | 21 (60.0%) | 149 (72.3%) |  |  |

| **Severity of staying asleep** |  |  | .52 | .90 |
| --- | --- | --- | --- | --- |
| None | 9 (25.7%) | 43 (20.9%) |  |  |
| Mild to very severe | 26 (74.3%) | 163 (79.1%) |  |  |
| **Severity of waking up early** |  |  | .83 | .90 |
| None | 11 (31.4%) | 61 (29.6%) |  |  |
| Mild to very severe | 24 (68.6%) | 145 (70.4%) |  |  |
| **Satisfaction with current sleep pattern** |  |  | .25 | .78 |
| Dissatisfied | 14 (40.0%) | 104 (50.5%) |  |  |
| Satisfied | 21 (60.0%) | 102 (49.5%) |  |  |
| **Age** (≥18), Mean (SD)^b^ | 47.66 (14.37) | 42.31 (12.82) | .03 | .54 |
| **Average number of hours asleep per day** (0-24), Mean (SD) | 6.90 (1.07) | 7.15 (1.90) | .45 | .90 |
| **Treatment Expectancy and Credibility/Acceptability Scale-Modified** (0-70), Mean (SD)^b^ | 51.63 (10.11) | 50.18 (10.35) | .44 | .90 |

^a^ Estimated in n(%) or Mean(SD)

^b^ *p*-values are based on independent t-test; the remaining is based on χ^2^

GAD-7 = Generalized Anxiety Disorder 7

PHQ-9 = Patient Health Questionnaire 9
