# Supplementary material for: A Fully Automated Self-help Biopsychosocial Transdiagnostic Digital Intervention to Reduce Anxiety and/or Depression and Improve Emotional Regulation and Well-being: Pre–Follow-up Single-Arm Feasibility Trial
Source: JMIR Form Res. 2023 May 30;7:e43385. doi: 10.2196/43385 (PMC10265433; doi:10.2196/43385)
Supplement: Multimedia Appendix 4 [file formative_v7i1e43385_app4.doc]

**Multimedia Appendix 4.**

Descriptive of outcomes at preintervention by clinical diagnostic presentation subgroup.

|  | **Anxiety**  **N=26**  **Mean (SD)** | **Depression**  **N=23**  **Mean (SD)** | **Comorbid**  **N=136**  **Mean (SD)** | **Non-clinical**  **N=56**  **Mean (SD)** | ***P*-value^a^** | **FDR** |
| --- | --- | --- | --- | --- | --- | --- |
| GAD-7^b^ | 12.35 (3.53) | 5.43 (1.67) | 15.03 (4.13) | 5.05 (1.86) | <.001 | <.001 |
| PHQ-9^c^ | 6.81 (2.25) | 12.91 (3.26) | 17.37 (4.94) | 5.05 (2.54) | <.001 | <.001 |
| K-6^d^ | 12.92 (2.87) | 15.04 (3.57) | 20.53 (4.73) | 10.63 (2.73) | <.001 | <.001 |
| DERS-36^e^ | 95.88 (19.37) | 92.04 (20.76) | 114.21 (21.49) | 83.93 (23.07) | <.001 | <.001 |
| MHC-SF^f^ | 38.42 (11.93) | 27.83 (11.01) | 24.93 (12.14) | 41.95 (12.22) | <.001 | <.001 |
| R-LOT^g^ | 13.08 (4.99) | 12.09 (3.60) | 10.01 (4.99) | 13.71 (4.63) | <.001 | <.001 |
| EQ-5D-3L Utility index | 0.77 (0.11) | 0.75 (0.11) | 0.61 (0.19) | 0.86 (0.14) | <.001 | <.001 |
| EQ-5D-3L Health rating | 71.46 (10.89) | 60.70 (16.16) | 54.86 (21.41) | 75.55 (12.69) | <.001 | <.001 |

^a^ *P*-values are based on ANOVAs.

^b^GAD-7: Generalized Anxiety Disorder 7

^c^PHQ-9: Patient Health Questionnaire 9

^d^K-6: Kessler 6.

^e^DERS-36: Difficulties in Emotional Regulation 36.

^f^MHC-SF: Mental Health Continuum—Short Form.

^g^R-LOT: Revised Life Orientation Test.
