# Supplementary material for: A Fully Automated Self-help Biopsychosocial Transdiagnostic Digital Intervention to Reduce Anxiety and/or Depression and Improve Emotional Regulation and Well-being: Pre–Follow-up Single-Arm Feasibility Trial
Source: JMIR Form Res. 2023 May 30;7:e43385. doi: 10.2196/43385 (PMC10265433; doi:10.2196/43385)
Supplement: Multimedia Appendix 5 [file formative_v7i1e43385_app5.doc]

**Multimedia Appendix 5.**

Sociodemographic and clinical characteristics of the sample preintervention by clinical diagnostic presentation subgroup.

| **Variables** | **Anxiety^a^**  **(N = 26)** | **Depression^a^**  **(N=23)** | **Comorbid^a^**  **(N = 136)** | **Nonclinical^a^**  **(N = 56)** | ***P*-value** | **FDR** |
| --- | --- | --- | --- | --- | --- | --- |
| **Gender identity** |  |  |  |  | .69 | .71 |
| Male | 10 (38.5%) | 8 (34.8%) | 44 (32.4%) | 23 (41.1%) |  |  |
| Female | 16 (61.5%) | 15 (65.2%) | 92 (67.6%) | 33 (58.9%) |  |  |
| **Country of birth** |  |  |  |  | .49 | .56 |
| Australia | 5 (19.2%) | 6 (26.1%) | 26 (19.1%) | 16 (28.6%) |  |  |
| Another country | 21 (80.8%) | 17 (73.9%) | 110 (80.9%) | 40 (71.4%) |  |  |
| **Country of residence** |  |  |  |  | .15 | .21 |
| Australia | 23 (88.5%) | 23 (100.0%) | 130 (95.6%) | 55 (98.2%) |  |  |
| Another country | 3 (11.5%) | 0 (0.0%) | 6 (4.4%) | 1 (1.8%) |  |  |
| **Aboriginal and Torres Strait Islander** |  |  |  |  | .45 | .54 |
| Neither Aboriginal nor Torres Strait Islander | 26 (100.0%) | 22 (95.7%) | 133 (97.8%) | 56 (100.0%) |  |  |
| Aboriginal or Torres Strait Islander | 0 (0.0%) | 1 (4.3%) | 3 (2.2%) | 0 (0.0%) |  |  |
| **Sexual orientation** |  |  |  |  | .95 | .95 |
| Heterosexual | 22 (84.6%) | 19 (82.6%) | 112 (82.4%) | 48 (85.7%) |  |  |
| Gay/Lesbian/Bisexual/Another/Rather not say | 4 (15.4%) | 4 (17.4%) | 24 (17.6%) | 8 (14.3%) |  |  |
| **Relationship status** |  |  |  |  | .00 | .004 |
| Single | 2 (7.7%) | 7 (30.4%) | 40 (29.4%) | 5 (8.9%) |  |  |
| Married | 18 (69.2%) | 9 (39.1%) | 46 (33.8%) | 40 (71.4%) |  |  |
| De-facto | 5 (19.2%) | 3 (13.0%) | 27 (19.9%) | 7 (12.5%) |  |  |
| Divorced/Separated/Widowed | 1 (3.8%) | 4 (17.4%) | 23 (16.9%) | 4 (7.1%) |  |  |
| **Education level** |  |  |  |  | .25 | .32 |
| Secondary | 2 (7.7%) | 3 (13.0%) | 28 (20.6%) | 3 (5.4%) |  |  |
| Vocational/TAFE | 4 (15.4%) | 6 (26.1%) | 27 (19.9%) | 15 (26.8%) |  |  |
| BA | 13 (50.0%) | 8 (34.8%) | 58 (42.6%) | 26 (46.4%) |  |  |
| Postgraduate | 7 (26.9%) | 6 (26.1%) | 23 (16.9%) | 12 (21.4%) |  |  |
| **Employment status** |  |  |  |  | .01 | .03 |
| Full-time | 15 (57.7%) | 8 (34.8%) | 54 (39.7%) | 26 (46.4%) |  |  |
| Part-time | 0 (0.0%) | 6 (26.1%) | 25 (18.4%) | 10 (17.9%) |  |  |
| Volunteer | 4 (15.4%) | 4 (17.4%) | 14 (10.3%) | 3 (5.4%) |  |  |
| Studying | 4 (15.4%) | 1 (4.3%) | 13 (9.6%) | 5 (8.9%) |  |  |
| Home duties/carer | 0 (0.0%) | 2 (8.7%) | 13 (9.6%) | 2 (3.6%) |  |  |
| Disability support | 0 (0.0%) | 1 (4.3%) | 8 (5.9%) | 0 (0.0%) |  |  |
| Retired | 1 (3.8%) | 0 (0.0%) | 7 (5.1%) | 1 (1.8%) |  |  |
| Another status | 2 (7.7%) | 1 (4.3%) | 2 (1.5%) | 9 (16.1%) |  |  |
| **Annual income** |  |  |  |  | .05 | .10 |
| <$40,000 | 5 (20.8%) | 6 (28.6%) | 49 (37.1%) | 6 (12.8%) |  |  |
| $40,000-<$80,000 | 9 (37.5%) | 11 (52.4%) | 49 (37.1%) | 19 (40.4%) |  |  |
| $80,000-<$120,000 | 6 (25.0%) | 2 (9.5%) | 25 (18.9%) | 15 (31.9%) |  |  |
| ≥$120,000 | 4 (16.7%) | 2 (9.5%) | 9 (6.8%) | 7 (14.9%) |  |  |
| **Residential location** |  |  |  |  | .56 | .62 |
| City/Metropolitan | 18 (69.2%) | 17 (73.9%) | 82 (60.3%) | 36 (64.3%) |  |  |
| Rural/Remote/Regional | 8 (30.8%) | 6 (26.1%) | 54 (39.7%) | 20 (35.7%) |  |  |
| **Accessed physical health services in last 4 weeks** |  |  |  |  | .14 | .21 |
| Never/Not current | 13 (50.0%) | 7 (30.4%) | 37 (27.2%) | 19 (33.9%) |  |  |
| Current use | 13 (50.0%) | 16 (69.6%) | 99 (72.8%) | 37 (66.1%) |  |  |
| **Accessed mental health services in last 4 weeks** |  |  |  |  | .11 | .17 |
| Never/not current | 14 (53.8%) | 7 (30.4%) | 43 (31.6%) | 24 (42.9%) |  |  |
| Current use | 12 (46.2%) | 16 (69.6%) | 93 (68.4%) | 32 (57.1%) |  |  |
| **Drink alcohol** |  |  |  |  | .00 | .01 |
| Never | 18 (69.2%) | 18 (78.3%) | 67 (49.3%) | 40 (71.4%) |  |  |
| Monthly or less | 8 (30.8%) | 5 (21.7%) | 69 (50.7%) | 16 (28.6%) |  |  |
| 2 - 4 times a month |  |  |  |  | .01 | .03 |
| 2 - 3 times a week | 13 (50.0%) | 13 (56.5%) | 62 (45.6%) | 31 (55.4%) |  |  |
| 4 or more times a week | 8 (30.8%) | 10 (43.5%) | 42 (30.9%) | 19 (33.9%) |  |  |
| **Use illicit drugs** | 4 (15.4%) | 0 (0.0%) | 5 (3.7%) | 2 (3.6%) |  |  |
| Never | 1 (3.8%) | 0 (0.0%) | 27 (19.9%) | 4 (7.1%) |  |  |
| I used too, but not in the last 12 months |  |  |  |  | .05 | .10 |
| No more than 12 times in the last 12 months | 2 (7.7%) | 6 (26.1%) | 26 (19.1%) | 4 (7.1%) |  |  |
| 2 - 4 times a month | 1 (3.8%) | 4 (17.4%) | 30 (22.1%) | 8 (14.3%) |  |  |
| 2 - 3 times a week | 6 (23.1%) | 1 (4.3%) | 21 (15.4%) | 12 (21.4%) |  |  |
| 4 or more times a week | 11 (42.3%) | 8 (34.8%) | 34 (25.0%) | 14 (25.0%) |  |  |
| **Smoke cigarettes** | 6 (23.1%) | 4 (17.4%) | 25 (18.4%) | 18 (32.1%) |  |  |
| Never have |  |  |  |  | .66 | .71 |
| I used too, but not anymore | 20 (76.9%) | 17 (73.9%) | 83 (61.0%) | 43 (76.8%) |  |  |
| Not regularly, but once in a while | 4 (15.4%) | 3 (13.0%) | 34 (25.0%) | 10 (17.9%) |  |  |
| Yes | 0 (0.0%) | 2 (8.7%) | 8 (5.9%) | 2 (3.6%) |  |  |
| **Use psychotropic medication** | 1 (3.8%) | 0 (0.0%) | 7 (5.1%) | 0 (0.0%) |  |  |
| No | 0 (0.0%) | 0 (0.0%) | 1 (0.7%) | 0 (0.0%) |  |  |
| Yes | 1 (3.8%) | 1 (4.3%) | 3 (2.2%) | 1 (1.8%) |  |  |
| **Do you feel you have enough social support / meaningful connections with other people** |  |  |  |  | .00 | .004 |
| Not at all | 0 (0.0%) | 1 (4.3%) | 17 (12.5%) | 2 (3.6%) |  |  |
| A little | 8 (30.8%) | 6 (26.1%) | 40 (29.4%) | 6 (10.7%) |  |  |
| Somewhat | 6 (23.1%) | 9 (39.1%) | 49 (36.0%) | 14 (25.0%) |  |  |
| Much | 8 (30.8%) | 6 (26.1%) | 19 (14.0%) | 17 (30.4%) |  |  |
| Very much | 4 (15.4%) | 1 (4.3%) | 11 (8.1%) | 17 (30.4%) |  |  |
| **Anxiety** |  |  |  |  | .00 | .004 |
| GAD-7 ≥8 | 26 (100.0%) | 0 (0.0%) | 136 (100.0%) | 0 (0.0%) |  |  |
| GAD-7 <8 | 0 (0.0%) | 23 (100.0%) | 0 (0.0%) | 56 (100.0%) |  |  |
| **Depression** |  |  |  |  | .00 | .004 |
| PHQ-9 ≥10 | 0 (0.0%) | 23 (100.0%) | 135 (99.3%) | 0 (0.0%) |  |  |
| PHQ-9 <10 | 26 (100.0%) | 0 (0.0%) | 1 (0.7%) | 56 (100.0%) |  |  |
| **Current Panic Disorder symptoms** |  |  |  |  | .00 | .01 |
| Never | 9 (34.6%) | 12 (52.2%) | 30 (22.1%) | 24 (42.9%) |  |  |
| Not now, but I used too | 4 (15.4%) | 1 (4.3%) | 17 (12.5%) | 13 (23.2%) |  |  |
| Sometimes | 8 (30.8%) | 7 (30.4%) | 47 (34.6%) | 12 (21.4%) |  |  |
| Yes | 5 (19.2%) | 3 (13.0%) | 42 (30.9%) | 7 (12.5%) |  |  |
| **Current Social Anxiety Disorder symptoms** |  |  |  |  | .00 | .01 |
| Never | 7 (26.9%) | 3 (13.0%) | 14 (10.3%) | 11 (19.6%) |  |  |
| Not now, but I used too | 2 (7.7%) | 2 (8.7%) | 9 (6.6%) | 8 (14.3%) |  |  |
| Sometimes | 11 (42.3%) | 11 (47.8%) | 49 (36.0%) | 29 (51.8%) |  |  |
| Yes | 6 (23.1%) | 7 (30.4%) | 64 (47.1%) | 8 (14.3%) |  |  |
| **Current Specific Phobia symptoms** |  |  |  |  | .07 | .13 |
| Never | 9 (34.6%) | 7 (30.4%) | 46 (33.8%) | 24 (42.9%) |  |  |
| Not now, but I used too | 2 (7.7%) | 3 (13.0%) | 10 (7.4%) | 8 (14.3%) |  |  |
| Sometimes | 7 (26.9%) | 9 (39.1%) | 34 (25.0%) | 19 (33.9%) |  |  |
| Yes | 8 (30.8%) | 4 (17.4%) | 46 (33.8%) | 5 (8.9%) |  |  |

| **Current Post Traumatic Stress Disorder symptoms** |  |  |  |  | .00 | .004 |
| --- | --- | --- | --- | --- | --- | --- |
| Never | 9 (34.6%) | 10 (43.5%) | 26 (19.1%) | 24 (42.9%) |  |  |
| Not now, but I used too | 5 (19.2%) | 4 (17.4%) | 20 (14.7%) | 13 (23.2%) |  |  |
| Sometimes | 2 (7.7%) | 6 (26.1%) | 34 (25.0%) | 14 (25.0%) |  |  |
| Yes | 10 (38.5%) | 3 (13.0%) | 56 (41.2%) | 5 (8.9%) |  |  |
| **Current Obsessive-Compulsive Disorder symptoms** |  |  |  |  | .00 | .004 |
| Never | 5 (19.2%) | 6 (26.1%) | 18 (13.2%) | 21 (37.5%) |  |  |
| Not now, but I used too | 2 (7.7%) | 5 (21.7%) | 9 (6.6%) | 10 (17.9%) |  |  |
| Sometimes | 7 (26.9%) | 7 (30.4%) | 46 (33.8%) | 20 (35.7%) |  |  |
| Yes | 12 (46.2%) | 5 (21.7%) | 63 (46.3%) | 5 (8.9%) |  |  |
| **Severity of falling asleep** |  |  |  |  | .04 | .10 |
| None | 10 (38.5%) | 9 (39.1%) | 30 (22.1%) | 22 (39.3%) |  |  |
| Mild to very severe | 16 (61.5%) | 14 (60.9%) | 106 (77.9%) | 34 (60.7%) |  |  |
| **Severity of staying asleep** |  |  |  |  | .41 | .51 |
| None | 7 (26.9%) | 6 (26.1%) | 24 (17.6%) | 15 (26.8%) |  |  |
| Mild to very severe | 19 (73.1%) | 17 (73.9%) | 112 (82.4%) | 41 (73.2%) |  |  |
| **Severity of waking up early** |  |  |  |  | .19 | .26 |
| None | 10 (38.5%) | 8 (34.8%) | 33 (24.3%) | 21 (37.5%) |  |  |
| Mild to very severe | 16 (61.5%) | 15 (65.2%) | 103 (75.7%) | 35 (62.5%) |  |  |
| **Satisfaction with current sleep pattern** |  |  |  |  | .00 | .004 |
| Dissatisfied | 4 (15.4%) | 11 (47.8%) | 91 (66.9%) | 12 (21.4%) |  |  |
| Satisfied | 22 (84.6%) | 12 (52.2%) | 45 (33.1%) | 44 (78.6%) |  |  |
| **Age** (≥18), Mean (SD)^b^ | 44.27 (11.63) | 44.74 (15.73) | 41.11 (12.95) | 46.66 (12.65) | .05 | .10 |
| **Average number of hours asleep per day** (0-24), Mean (SD)^b^ | 7.10 (0.85) | 7.13 (1.35) | 6.91 (1.80) | 7.61 (2.19) | .11 | .17 |
| **Treatment Expectancy and Credibility/Acceptability Scale-Modified** (0-70), Mean (SD)^b^ | 51.92 (9.63) | 48.09 (7.97) | 49.43 (11.01) | 52.96 (9.28) | .09 | .16 |

^a^ Estimated in n(%) or Mean(SD)

^b^ *P*-values are based on ANOVA; the remaining is based on χ^2^

GAD-7 – Generalized Anxiety Disorder 7

PHQ-9 = Patient Health Questionnaire 9
