# Supplementary material for: A Fully Automated Self-help Biopsychosocial Transdiagnostic Digital Intervention to Reduce Anxiety and/or Depression and Improve Emotional Regulation and Well-being: Pre–Follow-up Single-Arm Feasibility Trial
Source: JMIR Form Res. 2023 May 30;7:e43385. doi: 10.2196/43385 (PMC10265433; doi:10.2196/43385)
Supplement: Multimedia Appendix 6 [file formative_v7i1e43385_app6.docx]

**Multimedia Appendix 6.**

Study characteristics at preintervention (N=241).

|  | | Values  n(%) |
| --- | --- | --- |
| **Sex, n (%)** | | |
|  | Male | 85 (35.3) |
|  | Female | 156 (64.7) |
| **Country of birth, n (%)** | | |
|  | Australia | 188 (78) |
|  | Another country | 53 (22) |
| **Country of residence, n (%)** | | |
|  | Australia | 231 (95.9) |
|  | Another country | 10 (4.1) |
| **Aboriginal and Torres Strait Islander, n (%)** | | |
|  | Neither Aboriginal nor Torres Strait Islander | 237 (98.3) |
|  | Aboriginal or Torres Strait Islander | 4 (1.7) |
| **Sexual orientation, n (%)** | | |
|  | Heterosexual | 201 (83.4) |
|  | Lesbian | 7 (2.9) |
|  | Gay | 8 (3.3) |
|  | Bisexual | 19 (7.9) |
|  | Another or rather not say | 6 (2.5) |
| **Relationship status, n (%)** | | |
|  | Single | 54 (22.4) |
|  | In a relationship but not living together | 18 (7.5) |
|  | Defacto | 42 (17.4) |
|  | Married | 113 (46.9) |
|  | Divorced or separated | 13 (5.4) |
|  | Widowed | 1 (0.4) |
| **Education level, n (%)** | | |
|  | Year 12 or less | 36 (14.9) |
|  | Vocational (certificate, diploma) | 52 (21.6) |
|  | Bachelor’s degree or graduate certificate | 105 (43.6) |
|  | Master’s or doctoral degree | 48 (19.9) |

| **Employment status, n (%)** | | |
| --- | --- | --- |
|  | Employed full time | 111 (46.1) |
|  | Employed part time | 63 (26.1) |
|  | Volunteering | 40 (16.6) |
|  | Studying | 28 (11.6) |
|  | Home duties | 23 (9.5) |
|  | Carer | 11 (4.6) |
|  | Disability support | 10 (4.1) |
|  | Retired | 14 (5.8) |
|  | Another status | 6 (2.5) |
| **Annual income, n (%)** | | |
|  | <AUD $20,000 (US $13406) | 40 (16.6) |
|  | AUD $20,000-AUD $39,999 (US $13406-US $26812) | 26 (10.8) |
|  | AUD $40,000-AUD $59,999 (US $26813- US $40219) | 44 (18.3) |
|  | AUD $60,000-AUD $79,999 (US $40219- US $53625) | 44 (18.3) |
|  | AUD $80,000-AUD $99,999 (US $53626- US $67032) | 27 (11.2) |
|  | ≥ AUD $100,000 (US $67033) | 43 (17.8) |
|  | Prefer not to say | 17 (7.1) |
| **Residential location, n (%)** | | |
|  | City metro | 153 (63.5) |
|  | Regional | 65 (27) |
|  | Rural | 22 (9.1) |
|  | Remote | 1 (0.4) |
| **Accessed physical health services in last 4 weeks, n (%)** | | |
|  | Yes | 153 (63.5) |
|  | No | 88 (36.5) |
| **Accessed mental health services in last 4 weeks, n (%)** | | |
|  | Yes | 98 (40.7) |
|  | No | 143 (59.3) |
| **Drink alcohol, n (%)** | | |
|  | Never | 38 (15.8) |
|  | Monthly or less | 43 (17.8) |
|  | 2-4 times per month | 40 (16.6) |
|  | 2-3 times per week | 67 (27.8) |
|  | ≥4 per week | 53 (22.0) |
| **Use illicit drugs, n (%)** | | |
|  | Never | 163 (67.6) |
|  | Not in the last 12 months | 51 (21.2) |
|  | Monthly or less | 12 (5) |
|  | 2-4 times per month | 8 (3.3) |
|  | 2-3 times per week | 1 (0.4) |
|  | ≥4 per week | 6 (2.5) |
| **Smoke cigarettes, n (%)** | | |
|  | Yes | 32 (13.3) |
|  | Not regularly but sometimes | 11 (4.6) |
|  | Used too | 79 (32.8) |
|  | Never | 119 (49.4) |
| **Using psychotropic medication, n (%)** | | |
|  | Yes | 117 (48.5) |
|  | No | 124 (51.5) |
| **Do you feel you have enough social support/meaningful connections with other people, n (%)** | | |
|  | Not at all | 20 (8.3) |
|  | A little | 60 (24.9) |
|  | Somewhat | 78 (32.4) |
|  | Much | 50 (20.7) |
|  | Very much | 33 (13.7) |
| **Anxiety, n (%)** | | |
|  | GAD-7^a^ ≥8 | 162 (67.2) |
|  | GAD-7 <8 | 79 (32.8) |
| **Depression, n (%)** | | |
|  | PHQ-9^b^ ≥10 | 158 (65.6) |
|  | PHQ-9 <10 | 83 (34.4) |
| **Current panic disorder symptoms, n (%)** | | |
|  | Yes | 57 (23.7) |
|  | Some of the time | 74 (30.7) |
|  | Not now, but previously | 35 (14.5) |
|  | Never have | 75 (31.1) |

| **Current social anxiety disorder symptoms, n (%)** | | |
| --- | --- | --- |
|  | Yes | 85 (35.3) |
|  | Some of the time | 100 (41.5) |
|  | Not now, but previously | 21 (8.7) |
|  | Never have | 35 (14.5) |
| **Current specific phobia symptoms, n (%)** | | |
|  | Yes | 63 (26.1) |
|  | Some of the time | 69 (28.6) |
|  | Not now, but previously | 23 (9.5) |
|  | Never have | 86 (35.7) |
| **Current posttraumatic stress disorder symptoms, n (%)** | | |
|  | Yes | 74 (30.7) |
|  | Some of the time | 56 (23.2) |
|  | Not now, but previously | 42 (17.4) |
|  | Never have | 69 (28.6) |
| **Current obsessive-compulsive disorder symptoms, n (%)** | | |
|  | Yes | 85 (35.3) |
|  | Some of the time | 80 (33.2) |
|  | Not now, but previously | 26 (10.8) |
|  | Never have | 50 (20.7) |
| **Severity of falling asleep, n (%)** | | |
|  | None | 71 (29.5) |
|  | Mild | 82 (34) |
|  | Moderate | 48 (19.9) |
|  | Severe | 30 (12.4) |
|  | Very severe | 10 (4.1) |
| **Severity of staying asleep, n (%)** | | |
|  | None | 52 (21.6) |
|  | Mild | 86 (35.7) |
|  | Moderate | 63 (26.1) |
|  | Severe | 33 (13.7) |
|  | Very severe | 7 (2.9) |

| **Severity waking up too early, n (%)** | | |
| --- | --- | --- |
|  | None | 72 (29.9) |
|  | Mild | 69 (28.6) |
|  | Moderate | 66 (27.4) |
|  | Severe | 26 (10.8) |
|  | Very severe | 8 (3.3) |
| **Satisfaction with current sleep pattern** | | |
|  | Very satisfied, n (%) | 11 (4.6) |
|  | Satisfied, n (%) | 37 (15.4) |
|  | Moderately satisfied, n (%) | 75 (31.1) |
|  | Dissatisfied, n (%) | 87 (36.1) |
|  | Very dissatisfied, n (%) | 31 (12.9) |
|  | Age (≥18 years), mean (SD) | 43.1 (13.2) |
|  | Average number of hours asleep per day (0-24), mean (SD) | 7.1 (1.8) |
|  | Treatment Expectancy and Credibility/Acceptability Scale - Modified (0-70), mean (SD) | 50.4 (10.3) |

^a^GAD-7: Generalized Anxiety Disorder 7.

^b^PHQ-9: Patient Health Questionnaire 9.
