# Supplementary material for: A Fully Automated Self-help Biopsychosocial Transdiagnostic Digital Intervention to Reduce Anxiety and/or Depression and Improve Emotional Regulation and Well-being: Pre–Follow-up Single-Arm Feasibility Trial
Source: JMIR Form Res. 2023 May 30;7:e43385. doi: 10.2196/43385 (PMC10265433; doi:10.2196/43385)
Supplement: Multimedia Appendix 7 [file formative_v7i1e43385_app7.docx]

**Multimedia Appendix 7.**

Descriptive of intervention outcomes at the scheduled during intervention assessment time point by preintervention clinical diagnostic presentation subgroup.

|  | | During intervention | | | |
| --- | --- | --- | --- | --- | --- |
|  | | week 3 (n=66) | | week 5 (n=44) | |
|  | | n (%) | Mean (SD) | n (%) | Mean (SD) |
| **GAD-7^a^** | |  |  |  |  |
|  | Anxiety | 7 (10.6) | 8.00 (3.32) | 5 (11.4) | 5.20 (2.86) |
|  | Depression | 6 (9.1) | 5.33 (4.50) | 3 (6.8) | 5.00 (1.73) |
|  | Comorbid | 39 (59.1) | 10.10 (4.68) | 28 (63.4) | 7.29 (4.47) |
|  | Nonclinical | 14 (21.2) | 5.21 (4.00) | 8 (18.2) | 4.75 (2.55) |
| **PHQ-9^b^** | | | | | |
|  | Anxiety | 7 (10.6) | 4.29 (1.98) | 5 (11.4) | 5.00 (2.24) |
|  | Depression | 6 (9.1) | 8.00 (5.40) | 3 (6.8) | 9.67 (3.51) |
|  | Comorbid | 39 (59.1) | 12.62 (6.16) | 28 (63.4) | 9.86 (5.97) |
|  | Nonclinical | 14 (21.2) | 4.57 (3.34) | 8 (18.2) | 5.75 (4.10) |

^a^GAD-7: Generalized Anxiety Disorder 7.

^b^PHQ-9: Patient Health Questionnaire 9.
